# Multi-isotope analysis reveals that feasts in the Stonehenge environs and across Wessex drew people and animals from throughout Britain

**DOI:** 10.1126/sciadv.aau6078

**Published:** 2019-03-13

**Authors:** R. Madgwick, A. L. Lamb, H. Sloane, A. J. Nederbragt, U. Albarella, M. Parker Pearson, J. A. Evans

**Affiliations:** 1 School of History, Archaeology and Religion, Cardiff University, Cardiff CF10 3EU, UK.; 2 NERC Isotope Geosciences Laboratory, British Geological Survey, Keyworth, Nottinghamshire NG12 5GG, UK.; 3 School of Earth and Ocean Sciences, Cardiff University, Cardiff CF10 3AT, UK.; 4 Department of Archaeology, University of Sheffield, Sheffield S1 3NJ, UK.; 5 Institute of Archaeology, University College London, London WC1H 0PY, UK.; 6 School of Archaeology and Ancient History, University of Leicester, Leicester LE1 7RH, UK.; 7 Department of Classics and Archaeology, University of Nottingham, Nottingham, NG7 2RD, UK.

## Abstract

The great henge complexes of southern Britain are iconic monuments of the third millennium BCE, representing great feats of engineering and labor mobilization that hosted feasting events on a previously unparalleled scale. The scale of movement and the catchments that the complexes served, however, have thus far eluded understanding. Presenting the largest five-isotope system archeological dataset (^87^Sr/^86^Sr, δ^34^S, δ^18^O, δ^13^C, and δ^15^N) yet fully published, we analyze 131 pigs, the prime feasting animals, from four Late Neolithic (approximately 2800 to 2400 BCE) complexes to explore the networks that the feasts served. Because archeological evidence excludes continental contact, sources are considered only in the context of the British Isles. This analysis reveals wide-ranging origins across Britain, with few pigs raised locally. This finding demonstrates great investment of effort in transporting pigs raised elsewhere over vast distances to supply feasts and evidences the very first phase of pan-British connectivity.

## Teaser

Pan-British mobility is revealed in Neolithic Britain in the largest faunal multi-isotope study yet delivered in archeology.

## INTRODUCTION

The great Neolithic henge complexes of southern Britain are among the most iconic monuments of prehistoric Europe and have been the focus of research for as long as archeology has existed as a discipline (*1*). Their importance as ceremonial centers is evidenced by the resources invested in their construction and those expended during the feasts they hosted. These events were unrivaled in earlier periods and rarely paralleled even after the Roman invasion. Recent research has radically enhanced our understanding of the most famous of these complexes, Stonehenge, with the vast faunal assemblage of neighboring Durrington Walls providing evidence for great pig feasts that mainly occurred in winter (*2*). Stonehenge and Avebury are by far the most famous and well researched of the monumental complexes because of their imposing megalithic architecture. Other enclosures such as Mount Pleasant and Marden have been the focus of less research but were unquestionably centers for ceremonial activity and have also produced substantial pig-dominated faunal assemblages.

It has long been suggested that these events drew people from beyond the vicinity of the sites, but the volume and scale of movement remain poorly understood. There is no evidence for continental contact during this phase, and therefore, the presence of participants from mainland Europe can be excluded (*3*). Artefactual evidence, however, indicates contact between regions of the British Isles. Grooved ware, a distinctive style of pottery that is abundant at these complexes and central to the feasting events (*4*), is ubiquitous across Britain and Ireland between 2900 and 2200 BCE, providing evidence for connectivity (*5*).

Human remains would provide ideal material to examine connectivity, but these are scarce and most are cremated, meaning that they are unsuitable for most biomolecular analyses. Pigs are the prime animal used in feasting but have not previously been subjected to provenancing isotope analysis, as cattle have been favored as an animal more likely to have been moved under human control. In addition, porcine enamel was initially considered susceptible to diagenetic alteration, but research has since demonstrated that pigs retain biogenic strontium (*6*), thus releasing the potential of this important class of remains. The data presented here further support this research in showing great diversity in isotope values at single sites, demonstrating that diagenetic strontium from the burial environment has not homogenized the results. However, using pigs as a proxy for human movement is potentially problematic. Pigs are poorly suited to movement over distance (*7*) and, as the principal domesticate in Late Neolithic Britain, would have been far more easily procured near feasting centers than transported across Britain. Therefore, even if participants derived from wide-ranging locations, the animals they feasted on may have been locally raised. If pigs are established as nonlocal, then it is highly likely that they provide a good proxy for human movement. It cannot be completely discounted that preserved pork may have been transported by specialist producers, rather than animals being brought by feasting participants. However, this is very unlikely, as skulls and extremities are prevalent and these would be removed before preservation. In addition, there is no evidence for large scale, organized husbandry and preservation in Neolithic Britain. Although suitable for curing, pork spoils more rapidly than other meats (*8*), and its transport would have required a complex system of salting and/or smoking.

Previous biomolecular research on cattle provided tantalizing evidence for the existence of nonlocal animals at Durrington Walls, some of which must have come a substantial distance (*9*). This investigation used ^87^Sr/^86^Sr isotope analysis, which is useful for identifying nonlocal animals, but has greater interpretative potential when combined with other isotope proxies. In addition, the focus on cattle may provide an unbalanced view of connectivity, as pigs were far more common (*2*). Cattle values also have interpretative difficulties surrounding the effects of seasonally mobile husbandry regimes on isotope signals in their hypsodont teeth. Recent ^87^Sr/^86^Sr isotope research has also been undertaken on cremated human bone from Stonehenge itself (*10*). Data on 25 individuals also provided indications of nonlocal individuals, some posited to have potentially come from west Wales. These data provide more evidence for mobility but lack the resolution that might be achieved with multi-isotope proxies. In addition, as strontium is incorporated in bone over a period of several years, far longer than dental enamel, isotope values may provide a blended signal representing various areas in which individuals resided in the years before death, thus complicating interpretation.

This research uses a multi-isotope (^87^Sr/^86^Sr, δ^34^S, δ^18^O, δ^13^C, and δ^15^N) approach on 131 pigs from four Late Neolithic henge complexes to explore the scale and volume of movement and the catchments that they served. Multi-isotope studies using five systems are rare, and this represents the largest dataset of multiple (five) isotope methods yet applied to fauna in archeological research. Integrating provenancing data relating to geology (strontium, ^87^Sr/^86^Sr), climate (oxygen, δ^18^O), and coastal proximity (sulfur, δ^34^S), while taking account of the impact of diet on these values (δ^13^C and δ^15^N), means that this study has enhanced potential for exploring origins and identifying nonlocals than studies with fewer discriminants. A detailed description of methods used is provided in the Supplementary Materials.

### The sites

**Fig. 1:**
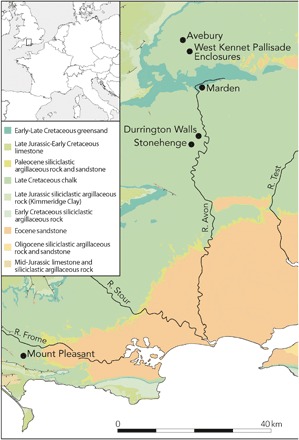
Location of the sites in central southern England (produced by K. Harding).

All sampled sites (Fig. 1) have produced substantial faunal and ceramic assemblages, strongly suggestive of feasting. All are founded on chalk lithology, with Marden being on the very fringes of the greensand. Durrington Walls is a large henge enclosure located approximately 3 km northeast of Stonehenge, Wiltshire. The peak period of activity centers on 2500 BCE, when it formed the largest known settlement in northwest Europe, perhaps housing more than 4000 people seasonally (*11*). Mount Pleasant is a henge enclosure in the Dorchester complex, situated 11 km from the coast in Dorset, and was a focus of activity in the mid-third millennium BCE (*12*). West Kennet Palisade Enclosures (WKPE) is part of the Avebury complex, Wiltshire, which comprises the world’s largest prehistoric stone circle (*13*). Situated 2 km southeast of Avebury, the site comprises two adjacent palisaded ditch enclosures of earlier date and Grooved ware settlement evidence dating to around 2500 to 2100 BCE (*14*). Marden, Wiltshire is located between Stonehenge and Avebury and is Britain’s largest henge monument, encompassing 14 ha (*15*). A major new program of archeological investigation is underway (*16*), but all samples derive from original excavations. Ceramic evidence and limited ^14^C dates suggest contemporaneity with Durrington Walls (*12*, *15*).

## RESULTS

**Fig. 2:**
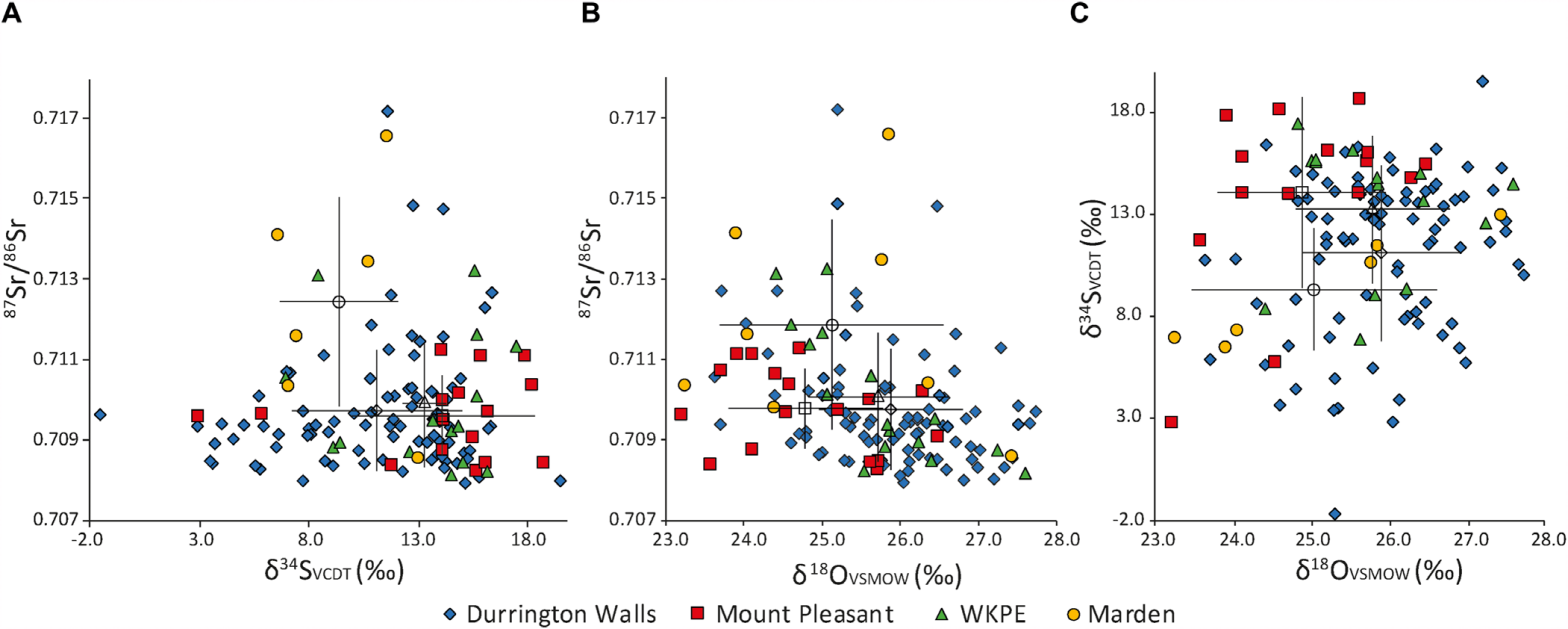
Bivariate plots showing isotope results for combinations of the three provenancing indices. Unshaded symbols represent means, and error bars denote 1 SD.

**Fig. 3:**
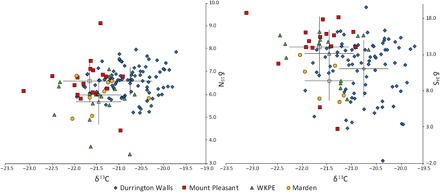
Bivariate plots showing δ^13^C and δ^15^N isotope results and δ^13^C plotted against δ^34^S. Unshaded symbols represent means, and error bars denote 1 SD.

**Fig. 4:**
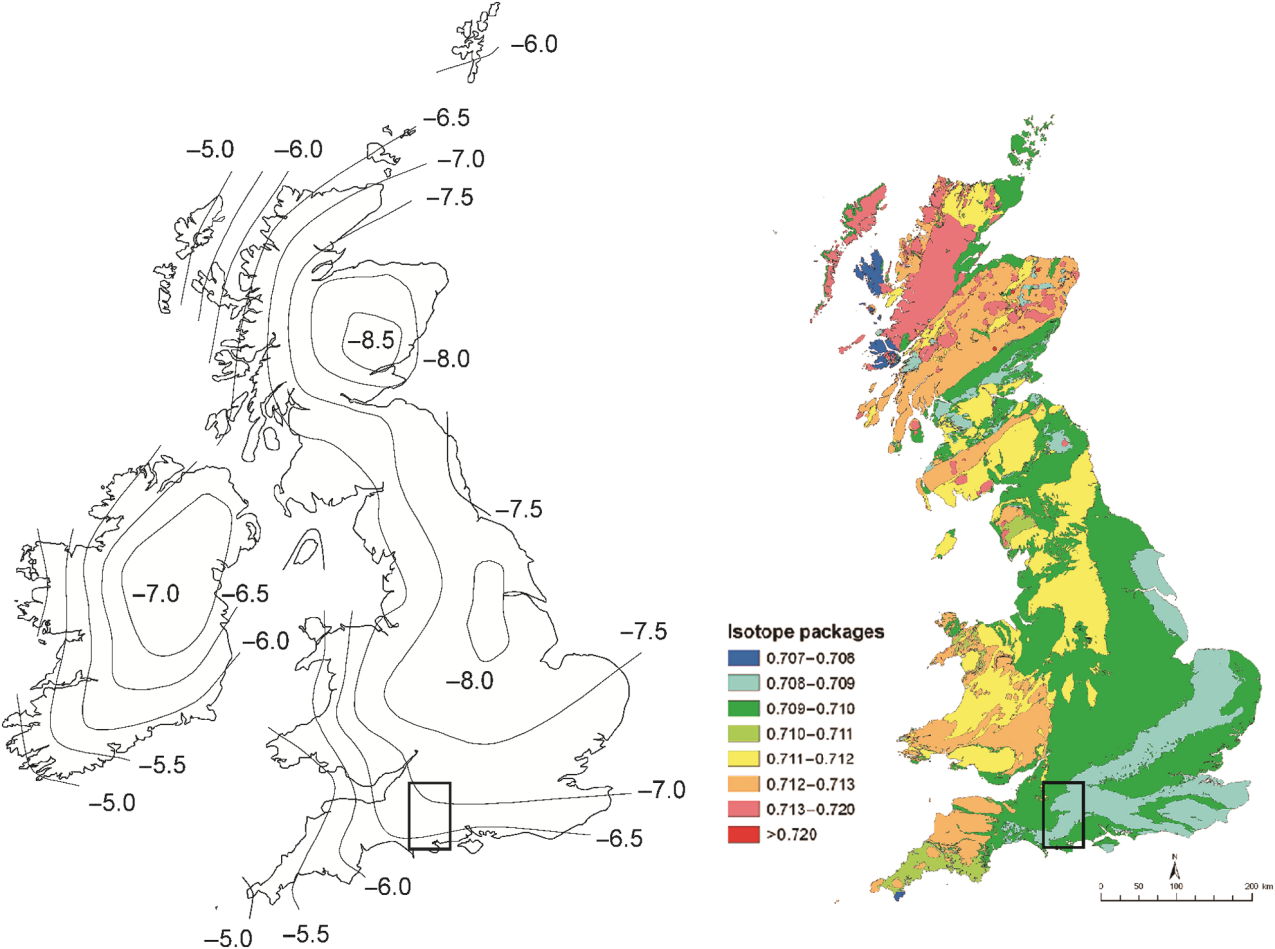
Map of δ^18^O_VSMOW_ in groundwaters of the British Isles (left) and Strontium (^87^Sr/^86^Sr) biosphere map of Great Britain (right). Inset: Region of site locations.

Isotope values were wide ranging (Table 1, Figs. 2 and 3, and tables S2 and S3). ^87^Sr/^86^Sr isotope results ranged from 0.7080 to 0.7172. These values cover all U.K. biosphere packages (Fig. 4) (*17*, *18*). Data from Durrington Walls show the greatest range (0.7080 to 0.7172), but the small Marden sample (*n* = 8) also has very diverse values (0.7086 to 0.7166). Marden has a higher median (0.7110) than other sites, with three samples providing highly radiogenic values [>0.7131, a threshold based on mapping research (*17*)]. The ranges at WKPE (0.7082 to 0.7133) and Mount Pleasant (0.7082 to 0.7113) are more restricted but are still very wide.

**Table 1:** Summary statistics for each site. IQR, interquartile range.

		**Durrington Walls**	**Mount Pleasant**	**WKPE**	**Marden**	**All**
^**87**^**Sr/**^**86**^**Sr**	*n*	89	18	16	8	131
	Median	0.7094	0.7097	0.7095	0.7110	0.7095
	Range	0.7080–0.7172	0.7082–0.7113	0.7082–0.7133	0.7086–0.7166	0.7080–0.7172
	IQR	0.0015	0.0017	0.0026	0.0034	0.0017
	Mean	0.7098	0.7098	0.7101	0.7120	0.7099
	1 SD	0.0016	0.0010	0.0017	0.0027	0.0017
**δ**^**18**^**O_VSMOW_ (‰)**	*n*	89	18	16	8	131
	Median	25.9	24.6	25.7	25.1	25.8
	Range	23.6–27.7	23.2–28.9	24.4–27.6	23.2–27.4	23.2–28.9
	IQR	1.27	1.58	1.23	1.97	1.38
	Mean	25.9	25.0	25.7	25.1	25.7
	1 SD	0.9	1.4	0.9	1.4	1.1
**δ**^**34**^**S_VCDT_ (‰)**	*n*	86	16	15	6	123
	Median	11.9	14.8	14.6	9.1	13.0
	Range	−1.6–19.6	2.8–18.8	6.9–17.5	6.5–13	−1.6–19.6
	IQR	5.7	2.0	5.4	4.2	5.8
	Mean	11.1	14.1	13.2	9.4	11.7
	1 SD	3.9	4.2	3.3	2.7	4.0
**δ**^**13**^**C_VPDB_ (‰)**	*n*	89	18	16	8	131
	Median	−20.7	−21.6	−21.4	−21.4	−21.0
	Range	−21.3–−19.7	−23.1–−20.9	−22.4–−20.6	−22.0–−20.3	−23.1–−19.7
	IQR	0.78	0.55	0.51	0.5	0.90
	Mean	−20.7	−21.7	−21.3	−21.4	−21.0
	1 SD	0.52	0.92	1.05	0.54	0.64
**δ**^**15**^**N_AIR_ (‰)**	*n*	89	18	16	8	131
	Median	6.6	6.6	6.0	6.0	6.5
	Range	5.2–8.0	4.5–9.1	3.4–7.2	5.1–6.8	3.4–9.1
	IQR	0.78	0.55	0.51	0.50	0.89
	Mean	6.6	6.8	6.2	5.7	6.5
	1 SD	0.63	0.92	1.10	0.65	0.79

δ^34^S isotope results also show a very wide range [−1.6 to 19.6 per mil (‰); median, 12.9‰]. Of the 123 samples that provided reliable replicate results, 45 show evidence of marine influence on their δ^34^S values (>14‰) (*19*), a high proportion considering that only one site is within 50 km of the coast. Durrington Walls is 56 km from the closest coastline and has the largest range (−1.6 to 19.6‰; median, 11.9‰), with 23 (27%) individuals having marine-affected values. Mount Pleasant, the only site that is close to the coast (11 km), also has a very large range (2.8 to 18.8‰; median, 14.8‰), with a clear tendency toward high values and 13 individuals (81%) showing marine influence. WKPE is much further from the closest coastline (74 km). Although the range of values at the site is not so wide (6.9 to 17.5‰), the median is high (14.6) and nine (60%) individuals show evidence of marine influence. Marden is the most distant site from the nearest shoreline (96 km) and has the lowest median value (9.1‰), with none of the individuals having marine-affected values. It also has the smallest range (6.5 to 13‰), but only six samples provided good replicate results.

δ^18^O isotope values ranged from 23.2 to 28.9‰. Pigs from Mount Pleasant are the most wide ranging (23.2 to 28.9‰; median, 24.6‰), although a single outlying high value skews the range, as the site has the lowest median. Durrington Walls (23.6 to 27.7‰; median, 25.9‰) and Marden (23.2 to 27.4‰; median, 25.1‰) also show wide variation, and samples from WKPE (24.4 to 27.4‰; median, 25.7‰) are only slightly more constrained.

δ^13^C isotope results range from −23.1 to −19.7‰ (median, − 21.0‰). There is considerable intersite variation, with Durrington Walls having significantly higher values than other sites (table S3). δ^15^N isotope results range from 3.4 to 9.1‰ (median, 6.5‰), but the range is skewed by a single high value (MP107), the only sample exceeding 8‰ and the same outlier as in the δ^18^O dataset. This sample was excluded from discussions of provenance, as its δ^18^O value must have been affected by foddering regimes. For example, the consumption of waste dairy products (e.g., from cheese-making) could explain these extreme values in both proxies. The intersite variation in δ^15^N results is less marked. δ^13^C and δ^15^N values are typical of terrestrial omnivores in Britain, and none of the outlying samples are characteristic of marine consumers (δ^13^C > −19‰; see table S3). Therefore, the input of marine-derived sulfur must be principally from sea spray rather than from consumption of marine produce (*19*), meaning that variation is driven by origins. This then allows us to use δ^34^S along with ^87^Sr/^86^Sr as an indicator of origin.

*K*-means cluster analysis was run on ^87^Sr/^86^Sr, δ^34^S, and δ^18^O for 122 samples (omitting eight poor δ^34^S duplicates and an outlying δ^18^O value affected by diet). This approach was undertaken to explore variation in the dataset, in terms of the number of clusters that could pertain to distinct isozones or regions and the balance of samples within these clusters. This method classifies samples into a predefined number of groups based on dissimilarity. Hierarchical cluster analysis was first performed to define the group number. This analysis provides a dendrogram showing the distance between each sample. The number of groups at different hierarchical dendrogram levels then informs the number of clusters in *K*-means analysis (fig. S1). Exploratory analysis led to a *K* = 24 model being most suited to the dataset (see Supplementary Materials for a detailed explanation of this process). Intracluster isotope ranges were sufficiently homogenous, with some being perhaps too broad in terms of their δ^34^S values (table S5). Reducing the number of clusters provided ranges in isotope values that were inconsistent with deriving from a single isozone.

This indicates a minimum of 24 isotopically distinct zones of provenance (or “isozones”). Isozones can be defined as areas with distinct natural or anthropogenic isotope baselines (*20*). Current methods do not have the capability to demonstrate the precise number of regions represented in the dataset, and these isozones must be interpreted with caution, as they do not necessarily represent far-flung, geographically disparate regions. This point is elaborated in Discussion.

## DISCUSSION

The Neolithic pig data are exceptionally wide-ranging, particularly in the three isotope proxies that are useful for investigating mobility (^87^Sr/^86^Sr, δ^34^S, and δ^18^O). The δ^13^C values are also wide-ranging, suggesting that the animals were raised in diverse landscape locations.

These data are strongly suggestive of a considerable volume of human-mediated movement. Isotope studies on faunal mobility remain relatively rare in Britain, and this is the first substantial multi-isotope dataset to be analyzed, but comparisons with other faunal data provide a useful starting point for the discussion.

### Comparative data

**Fig. 5:**
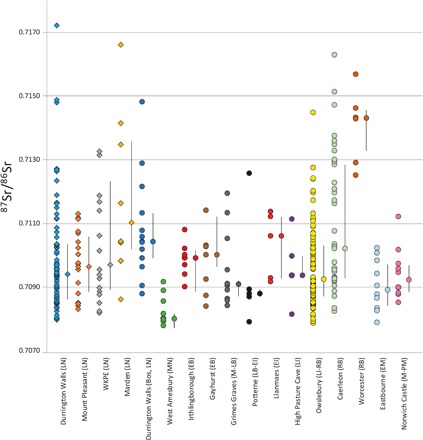
^87^Sr/^86^Sr isotope data for mainland British domestic fauna. After the Late Neolithic (LN) sites in this study (diamonds), sites are presented in approximate chronological order with periods abbreviated in parentheses [Middle Neolithic (MN), Early Bronze Age (EB), Middle-Late Bronze Age (M-LB), Late Bronze Age to Early Iron Age (LB-EI), Early Iron Age (EI), Late Iron Age (LI), Late Iron Age to Romano-British (LI-RB), Romano-British (RB), Early Medieval (EM), Medieval–Post-Medieval (M-PM)]. Median and IQR values are presented adjacent to the raw values for each site.

Of the isotope proxies useful for provenancing, only ^87^Sr/^86^Sr provides a reasonable comparative dataset. Published comparative data were considered if at least five animals of at least one domestic food species (i.e., excluding horses, dogs, and wild taxa) were analyzed at a site. Only mainland British sites were considered (with the inclusion of High Pasture Cave, Isle of Skye, because of its very close proximity to the mainland). A total of 13 sites fitted these criteria, comprising a comparative dataset of 220 animals [see Fig. 5, (data from *6*, *9*, *21*–*25*, *40*–*41*)]. Variation is discussed in terms of medians, ranges, and interquartile ranges (IQRs), as not all datasets are normally distributed.

The Durrington Walls pigs in this study have a larger range than all comparative sites. The next widest range comes from a sample of 37 cattle, caprines, and pigs from the Roman legionary fortress at Caerleon, South Wales (*21*). A large range might be predicted from fauna at Caerleon, as it is situated in an area of diverse geology and the legion might be expected to procure animals from further afield. However, the much smaller sample of only eight pigs from Marden, an area of much less complex lithology presented in this study, has a very similar range and IQR. WKPE and Mount Pleasant also have a very high IQR (ranked third and fifth of the 17 sites in the dataset). Considering the homogeneous geology across these sites, this provides clear evidence for wide catchments and a high proportion of animals being raised beyond the vicinity of the sites. Although Durrington Walls has the highest range and therefore also a wide catchment, the site’s IQR is more restricted. This does not necessarily mean a larger proportion of locally raised animals, and the wide variation in other isotope proxies shows that this is certainly not the case. However, in terms of ^87^Sr/^86^Sr, it suggests that a smaller proportion of individuals were coming from areas with very high or low values. This difference may, in fact, be an artifact of sample size, as all major biosphere packages in Britain are represented in the Durrington Walls dataset.

The most chronologically and geographically relevant comparisons are made with Late Neolithic cattle from Durrington Walls and the pigs and cattle from Middle Neolithic West Amesbury Farm, located only 3 km from Durrington Walls. Multiple samples were analyzed from the 13 cattle from Durrington Walls, but generally, intraindividual variation was limited (*9*), and sample means are therefore used for comparison. The median is higher, but the absolute range is markedly smaller than for the Durrington Walls pigs in this study, and the IQR is more restricted than all four sites in this study. Despite the small sample size, it would be expected that cattle would show greater variation because of their suitability to movement over distance. The nine animals from West Amesbury are in marked contrast with the Late Neolithic pigs presented here. They exhibit a very restricted range of 0.7078 to 0.7092, the smallest of all published British sites, suggesting largely local origins. Although the sample is limited, it indicates the greater degree to which the Stonehenge landscape drew in people and animals in the Late Neolithic in comparison to the Middle Neolithic. The only other study that is broadly comparable in terms of chronology focuses on the Early Bronze Age barrows of Irthlingborough and Gayhurst in central southern Britain (*22*). These barrows have evidence for hundreds of animals and show distinctive patterns of element representation, suggestive of people and animals converging on the sites. The sample was limited to only 15 cattle, but both sites had smaller absolute ranges and IQRs than all four sites in this study. The comparative datasets reinforce the exceptional degree of ^87^Sr/^86^Sr variation exhibited across the Late Neolithic pigs, providing strong evidence for humans and animals coming from wide-ranging locations in considerable numbers.

Highly radiogenic values are relatively rare in ^87^Sr/^86^Sr isotope studies in Britain. On the basis of current mapping, these values are the most distant from the local range of the Late Neolithic sites, both in terms of absolute values and biosphere location (see below). The comparative faunal dataset is dominated by southern British sites (the only exception being High Pasture Cave on the Isle of Skye) and therefore provides a useful comparison in terms of the prevalence of radiogenic values. Eight of the Late Neolithic pigs in this study provide highly radiogenic (>0.7131) ^87^Sr/^86^Sr values. These individuals derive from Durrington Walls (three), Marden (three), and WKPE (two) and represent 6.1% of the sample (table S2). Only 4 of the 13 comparative sites have radiogenic samples, comprising a total of 14 animals (6.3%). However, almost all of these are from sites with local radiogenic biosphere values [Caerleon (*21*) and Worcester (*23*)]. If these sites are excluded, then only 2 (1.1%) of the 177 remaining comparative faunal samples provide radiogenic values—cattle from Owslebury (*24*) and Durrington Walls (*9*). This demonstrates how rare animals with high values are at southern British sites that do not have a locally radiogenic biosphere. Marden stands out as having a particularly high median value (0.7110), with only Roman Worcester (which has a locally radiogenic biosphere), being higher (0.7143). Although the Marden sample is small, these results demonstrate that the site drew in animals and people from afar.

Comparative data for δ^18^O are much sparser. Only four sites from mainland Britain, Early Bronze Age Irthlingborough and Gayhurst, Middle-Late Bronze Age Grimes Graves and Roman Worcester, provide useful comparators (*23*, *25*, *26*). A total of 30 animals, all cattle, have δ^18^O data, but all sampling was undertaken for the purposes of seasonality studies and is therefore an imperfect comparative dataset. These 320 incremental data points are, however, useful for establishing potential ranges. Wider variation would be expected in cattle, as unlike pigs, hypsodont molars exhibit strongly seasonally variable signatures. The Late Neolithic pigs are more variable, but this is skewed by the single Mount Pleasant sample (MP107) with a very high value thought to relate to an anomalous diet. The 320 samples show an absolute range of 5‰, compared to 5.7‰ in the pig dataset (4.7‰ if MP107 is excluded). The IQR is larger in the cattle dataset (1.6‰ compared to 1.4‰ for pigs), as would be expected given the impact of seasonal variability. Comparing incremental δ^18^O values from seasonally variable cattle with single samples from pigs is not ideal, but the fact that absolute variation is comparable provides further support for interpretation of wide-ranging origins for the pigs at all sites, as seasonal variation cannot be responsible for differences. Note that the small Marden sample has the largest IQR of all the sites (2.0‰), further emphasizing the particularly diverse origins at this site despite its limited sample size.

Comparative domestic faunal δ^34^S isotope data are limited to only Irthlingborough and Gayhurst (*22*). This sample is very limited (*n* = 10 for each site) but exhibited a smaller range (10‰) than each of the Late Neolithic sites except Marden and a smaller IQR (3.6‰) than all sites except Mount Pleasant. The range of Durrington Walls samples (21.2‰) was more than double that of the comparative sample. However, note that half of the Gayhurst and Irthlingborough animals had negative values, in marked contrast to the Late Neolithic pigs that comprised only one negative value from 123 samples. This suggests that certain parts of the United Kingdom are poorly represented or absent in the Late Neolithic pig dataset. Negative values are often produced in waterlogged contexts, and therefore, it seems that few pigs were raised in these environments. Overall, the analysis of comparative data provides clear evidence for wide-ranging variation in isotope values in the Late Neolithic sample, indicative of animals and humans converging on the site from numerous different regions, some from a substantial distance away. This contrasts with the evidence from the flint and ceramic assemblages from Durrington Walls, which are overwhelmingly consistent with a local origin (*27*). The sarsen stones are likely to derive from the Marlborough Downs, approximately 32 km to the north, providing evidence for intraregional movement of substantial materials, but the inner ring and horseshoe of bluestones, deriving from West Wales, provides the best evidence for long-distance movement (*28*).

### Intersite variation

In general, isotope values for the five proxies were very diverse across all sites. However, some marked intersite variation was observed. Pairwise Mann-Whitney *U* (MWU) tests of difference were undertaken for each isotope method to assess significant differences in values between sites. Bonferroni-adjusted significance levels (0.0083) were used to negate the risk of type 1 error. This uses a conservative approach to statistical analysis and should avoid false-positive results. Differences that were significant at the standard (0.05) level were also noted but were not relied on in the interpretation. ^87^Sr/^86^Sr isotope results were very variable across all sites, and therefore, there was little evidence for intersite variation. Marden produced more radiogenic values than Durrington Walls, but this was only significant at the standard (0.05) level (*P* = 0.011; MWU, 166.5). The variable results and lack of strongly significant differences provide no evidence that these sites supported different regional networks. They all had large catchments, drawing in people and animals from various locations, although the range of values is more limited at Mount Pleasant.

δ^34^S showed a greater intersite variation, with Mount Pleasant having higher values than both Durrington Walls and Marden (significant at 0.0083). This demonstrates that pigs raised in coastal locations are more common at Mount Pleasant. This is perhaps unsurprising, as the site is located near the coast and it hints that Mount Pleasant supported a more local network than other sites. However, δ^18^O isotope results indicate that this is not the case (see below). WKPE pigs also comprise higher δ^34^S isotope values than those in Durrington Walls and Marden (significant at standard level). Mount Pleasant was again an outlier in terms of δ^18^O, having significantly lower values than Durrington Walls and WKPE, the latter only significant at the standard level. This difference is surprising because, contrary to the sulfur data, this is inconsistent with a more local origin for pigs at Mount Pleasant. There is a well-established east-to-west gradient of increasing δ^18^O isotope values [Fig. 4, map of groundwaters modified from (*29*)]. Mount Pleasant is the most westerly located of the sites and therefore would be expected to have higher values if supporting a local network. The significantly lower values are suggestive of an easterly origin for a substantial number of the pigs. These δ^18^O values, combined with the high δ^34^S values and the relatively low range of ^87^Sr/^86^Sr values compared to the other sites, are suggestive of a concentration of pigs deriving from the east coast of England. This is unlikely to represent a single community, but rather people drawn from various locations across this large area, as cluster analysis shows Mount Pleasant samples occupying a range of clusters. Other sites show greater variation and appear to support more widespread networks.

Interesting intersite variation was also observed in terms of δ^13^C and δ^15^N isotope values. The most striking pattern is that Durrington Walls pigs have significantly higher δ^13^C values than all other sites [Mount Pleasant: significance, <0.001 (MWU, 167.5); WKPE: significance, <0.001 (MWU, 259); Marden: significance, 0.002 (MWU, 130)]. This is difficult to explain, as the provenancing isotope proxies indicate that these pigs must have come from diverse locations, so this cannot relate to feeding in the same landscape. Therefore, many of the pigs at Durrington Walls must have been raised in a similar manner but in different locations. Higher δ^13^C values could result from a greater reliance on forest resources (*30*). Archeological evidence suggests larger feasting events at Durrington Walls than at the other sites. Perhaps disparate communities were raising more pigs for these events than could be easily sustained on resources immediately available around settlements. Consequently, it may have been necessary to drive pigs to woodland to fatten them on the abundant resources (e.g., nuts, acorns, and fungi) in these ecosystems. This could explain the higher δ^13^C values, but it cannot be entirely ruled out that the animals were fed a prescribed diet to be deemed fit to be feasted on at these ceremonial events.

δ^15^N results were far more homogeneous in the context of British fauna, with only a single outlier (MP107) having a value higher than 8‰. This was particularly clear at WKPE, which had the lowest mean value and significantly lower δ^15^N values than those at Durrington Walls (significance, 0.005; MWU, 400) and Mount Pleasant [significant only at the 0.05 level (0.049); MWU, 87]. This indicates that pigs were overwhelmingly raised on plant resources across all sites and that the exploitation of animal protein (e.g., meat, dairy produce, and feces) was particularly rare at WKPE. This is a common feature in Neolithic pig husbandry (*30*). Evidence suggests that norms of practice dictated that pigs should be raised on herbivorous diets in Late Neolithic Britain. This makes MP107, the pig with by far the highest δ^15^N and δ^18^O values, all the more interesting in having been raised on animal protein, potentially waste products from dairy processing, for a period during their development.

### Identifying nonlocal animals

The wide-ranging signatures in all isotope proxies demonstrate that many animals were not raised in the vicinity (within approximately 50 km) of the sites. However, identifying specific nonlocal animals is not straightforward. Only ^87^Sr/^86^Sr isotope analysis provides a reliable means for defining a local signal. All four sites lie on chalk lithologies. While some zones of the ^87^Sr/^86^Sr biosphere map [Fig. 4, strontium biosphere map modified from (*18*)] represent extrapolations from limited datasets, the local range for Wessex chalk is well understood. Evans *et al.* (*18*) documented 31 analyses of plant and dentine samples from chalk lithologies in Britain and designated the chalk range as between 0.7077 and 0.7087, as is supported by the analysis of seven plants from the landscape surrounding Durrington Walls (*9*). Local ranges for sulfur isotopes are more difficult to determine. Nehlich’s (*19*) global review of sulfur isotope research demonstrates that biosphere values of <14‰ can be expected at inland locations (>50 km from the coast). A review of British data indicates that the minimum coastal (approximately <30 km from the coast) biosphere value that can be expected is 8‰ (*17*). Therefore, individuals consistent with local origins can be defined as >8‰ for Mount Pleasant and <14‰ for the other sites. Using these local parameters, it is clear that the vast majority of animals from all four sites are not consistent with being locally raised, with only 13 (15%) from Durrington Walls, 4 (22%) from Mount Pleasant, none from WKPE, and 1 (13%) from Marden being consistent with a local origin. It is certain that this in fact exaggerates the number of local individuals, as the wide variation in both δ^34^S and δ^18^O values in each site’s “local” sample (e.g., 25.0 to 27.0‰ and 3.4. to 13.8‰, respectively, for Durrington Walls) is too diverse to derive from a single location.

This characterization of local individuals relates to the environs of the sites, but it is worth considering how this relates to the broader surrounding region. While the conservative local ranges for δ^34^S are unlikely to vary over a much larger area (approximately 30-km radius, with the possible exception of inland areas close to Mount Pleasant), different lithologies are present, and therefore, biosphere ^87^Sr/^86^Sr ranges would be wider at a regional level. New mapping data (*17*) indicate that values as high as 0.7113 could be attained on the greensand of the Vale of Pewsey and the Jurassic coast (<15 km from all sites). Extending the regional range accordingly would increase the number of individuals potentially raised in the surrounding region considerably to 59 (66%) at Durrington Walls, 18 (100%) at Mount Pleasant, 5 (31%) at WKPE, and 4 (50%) at Marden. However, the diverse range of δ^18^O and δ^34^S values also demonstrates that these animals were raised across very varied locations beyond the surrounding landscape (e.g., 23.7 to 27.7‰ and −1.6 to 13.9‰, respectively, for Durrington Walls), and therefore, this unquestionably greatly exaggerates the number of individuals that could derive from the surrounding region. This wide local range encompasses most of Britain, and therefore, these animals could come from any number of other regions. In addition, lithological zones that can produce these higher ^87^Sr/^86^Sr values are very restricted in the south, and therefore, it is unlikely that such a high proportion of animals were raised in these areas. Overall, the data demonstrate that most of the animals were not raised in the vicinity of the sites.

Further refinement is not currently possible, as δ^34^S mapping remains relatively low resolution, and is useful principally in differentiating coastal and noncoastal zones, and δ^18^O isotope data cannot be incorporated, as establishing expected local pig enamel values (rather than drinking water values) is very challenging. Fractionation effects between drinking water and carbonate and phosphate in tissues of different taxa are poorly understood, and the efficacy of equations for converting tissue values to meteoric water is variable (*31*). Consequently, exploring provenance through a direct comparison of δ^18^O values is advisable (*31*), especially in pigs where the relationship with drinking water is complex (*32*).

### Diverse origins

The cluster analysis provides a useful starting point for exploring the origins of the animals and indicates a minimum of 24 groups. Some groups in the *K* = 24 model are likely to be too heterogeneous to map onto single-biosphere zones (e.g., clusters 6 and 18; see table S5), and therefore, this figure may be conservative. However, these clusters cannot be mapped onto regions, and some could represent isozones that are in close proximity. In some instances, different clusters may be distinct in terms of the three isotope proxies but could represent adjacent regions, perhaps even comprising areas where the same community lived, for example, on two sides of a geological boundary. By contrast, it is certain that some very disparate regions of Britain are indistinguishable using the three isotope proxies. Therefore, just as two clusters could represent adjacent isozones that are geographically indistinct, one cluster could comprise several divergent geographical areas that are isotopically indistinct. This is highly likely to be the case for some of the well-represented clusters (e.g., cluster 13; see table S5). Overall, the number of distinct clusters and the wide range of values in the three provenancing isotope proxies provide strong evidence for wide-ranging origins.

Isotope data are generally most useful for eliminating potential areas of origin rather than for pinpointing locations. However, the diversity of values makes it difficult to exclude any areas of origin in Britain and Ireland from the dataset. The scale, volume, and diversity of movement are indisputable. Many regions are represented, most of the animals (and, by inference, humans) were not local to the sites, and some had traveled vast distances. ^87^Sr/^86^Sr isotope analysis is arguably the most informative in terms of exploring potential origins. Eight samples (three from Durrington Walls, three from Marden, and two from WKPE) have highly radiogenic ^87^Sr/^86^Sr values (>0.7131; see table S8). These values are very rare in the British biosphere and are therefore worthy of further comment.

Current mapping (*17*) indicates that only very small pockets of the biosphere in England and Wales can produce values such as these (isolated areas of southeast, southwest, and northwest Wales, the Malvern Hills in central western England, and the Lake District in northwest England). In any case, these areas are only likely to be able to account for the lowest three values (<0.7136): two from WKPE and one from Marden. On the basis of the current mapping, the other five animals, with values ranging from 0.7141 to 0.7172, are likely to derive from Scotland, where much larger areas of radiogenic geology exist. All of these areas of origin represent monumental distances for livestock, especially pigs, to be moved in prehistory. The animals with high values certainly derive from different locations, as they have wide-ranging ^87^Sr/^86^Sr (0.7131 to 0.7172‰), δ^34^S (6.5 to 15.6‰), and δ^18^O (23.9–26.5‰) results, with inland and coastally raised individuals in evidence. Higher-resolution mapping may identify more areas that can yield these radiogenic values. However, it is highly unlikely that any will be found in the large swathes of comparably recent geology that dominate southern, central, and eastern England.

On the basis of current mapping data, it is not possible to define origins with confidence, even when using multi-isotope proxies. Equifinality remains a hurdle to interpretation, as some areas may not be distinguishable, even when using five isotope proxies. The multi-isotope method can, however, refine provenance by discounting more potential source areas and by demonstrating the diversity of areas of origin. With improved mapping data and, potentially, the addition of other isotope proxies [e.g., Pb (*33*)], the identification of origins may be possible in the future.

### Archeological implications

The evidence for wide-ranging origins and the movement of pigs over prodigious distances demonstrates that complexes were not just power bases in the heartland of regional groups, at which feasting events acted to unify a disparate, yet broadly local populace, nor were these sites of reciprocal feasting, where alliances between neighboring groups were forged and consolidated. These centers were lynchpins for a much greater scale of connectivity, involving disparate groups from across Britain. Data indicate a volume and scale of movement not previously evidenced (*9*, *10*).

Transporting pigs over even modest distances across the Neolithic landscape would have required considerable effort. Ethnoarcheological examples of the movement of pigs over distance are very sparse. Rare examples (>100 km) have been cited in Greece (*34*) and Sardinia (*35*) but are exceptional compared to the common movement of caprines and cattle. Arguably, the most startling finding is the effort that participants invested in contributing pigs that they themselves had raised. Pigs were the principal domesticate and, therefore, procuring them in the vicinity of the feasting sites would have been relatively easy. Pigs are not nearly as well suited to movement over distance as bovids, and transporting them, either slaughtered or on the hoof, over hundreds or even tens of kilometers would have required a monumental effort. This suggests that prescribed contributions were required and that rules dictated that offered pigs must be raised by the feasting participants, accompanying them on their journey, rather than being acquired locally. It is likely that maritime and riverine transport played an important role in these networks. This mode of transporting pigs has been used from prehistory to the present in Indonesia and Papua New Guinea (*7*, *36*). Driving pigs overland would have represented a formidable challenge. Whatever mode of movement was used, a vast investment of effort would have been required. The transport of bluestones to Stonehenge from the Preseli Hills in Wales (*28*) demonstrates the challenges that communities overcame at these monumental complexes. There has been little research on mobility within Late Neolithic Britain, and the work on the bluestones provides the best evidence for interregional links. Note that some pigs have isotope values consistent with deriving from this location, further supporting the links between the Stonehenge landscape and West Wales. However, equifinality remains an interpretative issue, and it is plausible that these animals could also come from other locations (potentially southern Scotland or southwest England).

Results demonstrate that the Late Neolithic was the first phase of pan-British connectivity, with the scale of population movement across Britain arguably not evidenced in any other phase in prehistory. These long-distance networks were sustained by the movement not only of people but also of livestock. The complexes represent lynchpins for these networks, and it is not only the famous megalithic centers of Stonehenge and Avebury that were major foci. All four sites show long-distance connectivity, and there is no indication that they served different networks; all drew people and animals from across Britain. After more than a century of debate concerning the origins of people and animals in the Stonehenge landscape, these results provide clear evidence for a great volume and scale of intercommunity mobility in Late Neolithic Britain, demonstrating a level of interaction and social complexity not previously appreciated.

## MATERIALS AND METHODS

### Samples

Pigs were selected for analysis, as they were the principal domesticate at all four sites and were most likely to represent the remains of feasts. Samples for each site are listed in table S1. The sampling strategy was described separately for each isotope method below. All samples were confidently assigned to Late Neolithic deposits, although precise contextual information was unavailable for Mount Pleasant (remains were boxed by phase). Mandibles with a first molar that was in wear were favored for analysis. This would likely provide comparable snapshots of early life origins, and teeth in wear will have (at the very least) neared completion of the mineralization process. This sampling strategy was possible for all Durrington Walls samples, but a degree of flexibility was required for the other sites. The principal criteria for other sites were that remains could be confidently assigned to a Late Neolithic phase on the basis of radiocarbon dates, stratigraphy, and associated Grooved ware pottery and that teeth were in wear. Because of less detailed paper archives than for Durrington Walls, many samples had to be excluded because of the provenance being less than secure. Therefore, sample numbers are far smaller for the other sites, and in several instances, different teeth have been sampled. However, every effort was made to analyze a part of the tooth that developed at approximately the same period in the animal’s development (see details on sampling for each isotope method below). It was not possible to always sample the same-sided elements, but the variation in isotope results demonstrates that repeat sampling of the same individual was not a problem in this study. The vast majority of remains were recovered from middens, large spreads of material culture presumed to result from feasting. In some instances, remains from pits, ditches, and postholes were also analyzed. Care was taken to avoid the sampling of wild boar, and large outliers were avoided. Chronologically later samples from WKPE were also carefully avoided. More details on specific sampling strategies for each isotope system are provided in the Supplementary Materials.

### Background

δ^34^S isotope values from bone collagen vary according to geology, diet, and coastal proximity. High values (>14‰) invariably indicate an individual raised close (<50 km) to the coast, because of sea spray affecting the food chain, as long as substantial marine input in feeding can be discounted (*19*). The method has proved useful in exploring the movement and management of animals as part of a multiproxy approach (*37*). ^87^Sr/^86^Sr isotope analysis of dental enamel is a long-established method that principally provides evidence of the geological region where an individual was raised (*6*).

δ^18^O isotope values in tissues principally derive from ingested water and reflect climate, varying both geographically (*29*) and seasonally (*38*). The effects of seasonal climatic variation are limited in pig molars (*38*). This is likely to result from short crowns and rapid development, and therefore, seasonal variation is highly unlikely to affect values in the first molars sampled in this study. However, results are useful for identifying origins. Dietary variation can also affect δ^18^O isotope values (*31*), a point which is discussed in relation to a single sample (MP107). Carbon (δ^13^C) and nitrogen (δ^15^N) isotope analyses of collagen are used for reconstructing past diets and have proved useful in investigating foddering and management in pigs (*30*). It also provides important baseline data for interpreting ^87^Sr/^86^Sr, δ^34^S, and δ^18^O isotope results by establishing marine input in feeding and by identifying samples for which other isotope proxies might be affected by foddering regimes. *K*-means cluster analysis was applied to the provenancing isotope datasets (^87^Sr/^86^Sr, δ^34^S, and δ^18^O) to group similar samples and to explore the range of geographically defined isotope packages represented in the dataset.

### Sample preparation and analysis

Lower first molar enamel (^87^Sr/^86^Sr, δ^18^O) and associated mandibular bone (δ^13^C, δ^15^N, and δ^34^S) were sampled (table S1). In some instances, it was necessary to sample other teeth. All ^87^Sr/^86^Sr, δ^18^O, and δ^34^S isotope analyses were undertaken at the Natural Environment Research Council (NERC) Isotope Geosciences Laboratory (NIGL), Keyworth. The enamel surface of each tooth was abraded to a depth of >100 μm using a tungsten carbide dental burr, and the residue was discarded. Thin enamel slices (1 to 2 mm in breadth) were then cut from the tooth using a flexible diamond-edged rotary wheel attached to an Argofile PS220 precision drill. All surfaces were mechanically cleaned with a diamond burr to remove adhering dentine. For ^87^Sr/^86^Sr isotope analysis, samples were transferred to a clean (class 100, laminar flow) area, washed in high-purity acetone, cleaned ultrasonically, dried, and weighed into Teflon beakers. The samples were mixed with ^84^Sr tracer solution and dissolved in Teflon-distilled 8 M HNO_3_. Strontium was collected using Dowex resin columns. Strontium was loaded onto a single Re filament with TaF (Tantalum emitter), and the isotope values and concentrations were determined by thermal ionization mass spectrometry using a Thermo Triton multicollector mass spectrometer. The international standard for ^87^Sr/^86^Sr, NBS987, gave a value of 0.710255 ± 0.000012 (*n* = 68, 2σ) during analysis. Blank values were in the region of 100 pg. The carbonate fraction of dental enamel was sampled for δ^18^O isotope analysis. In all instances, the same tooth was sampled as for ^87^Sr/^86^Sr isotope analysis. Clean enamel was powdered, weighed (2 mg ± 0.5 mg), loaded into glass vials, sealed with septa, and transferred to a hot block (90°C) on a MultiPrep system. Vials were evacuated, and four drops of anhydrous phosphoric acid were added. The resultant CO_2_ was collected cryogenically for 15 min and transferred to a GV IsoPrime dual inlet mass spectrometer. The resultant isotope values are reported as per mil (‰) normalized to the Vienna Pee Dee belemnite (VPDB) scale using an in-house carbonate reference material (KCM: Keyworth Carrara Marble) calibrated against an NBS19-certified reference material. The δ^18^O carbonate values were then converted into the Vienna standard mean ocean water (VSMOW) scale [VSMOW = 1.0309 × δ^18^O VPDB + 30.91 (*39*)]. The batch reproducibility for δ^18^O KCM was ±0.09‰ (1σ). A total of 39 samples were analyzed in duplicate, providing a mean SD (1σ) for replicate samples of 0.13‰.

The collagen extraction protocol followed a modified Longin method. Bone or dentine (approximately 0.5 g for bone and 0.1 to 0.4 g for dentine) was cleaned using a diamond burr, demineralized in 8 ml of 0.5 M HCl at 4°C, and gelatinized in HCl (pH 3) at 70°C for 48 hours. The supernate was collected using an 8-μm Ezee filter and transferred to polypropylene tubes for freeze-drying. For δ^34^S isotope analysis, V_2_O_5_ was added to aid combustion. Samples were processed in duplicate as a minimum, but in some instances, four replicates were analyzed to ensure reliable repeats. There was one exception to this, sample WK120, which produced insufficient collagen for replicate analysis. Isotope ratios were measured by continuous flow–elemental analysis–isotope ratio mass spectrometry. δ^34^S isotope analysis was undertaken at NIGL, Keyworth. δ^13^C and δ^15^N isotope analyses were undertaken at Cardiff University, NIGL (Keyworth), and the University of Cambridge. Details of the instrumentation, standards, and reproducibility are provided in the Supplementary Materials. Collagen δ^13^C, δ^15^N, and δ^34^S isotope values are reported in per mil (‰) relative to VPDB, AIR, and VCDT (Vienna Canyon Diablo Troilite) standards, respectively.

### Statistical analysis

Cluster analysis was undertaken using IBM SPSS Statistics 20. Variables were first standardized to produce *z* scores so that each would have equal weighting in the models. Only samples that provided valid data for all three isotope provenancing indices were included in the analysis (*n* = 122). Eight samples that provided poor δ^34^S replicates were omitted, and the single sample from Mount Pleasant (MP107) with anomalous δ^15^N and δ^18^O values (that can be explained by an unusual diet) was also omitted as this would alter the model for reasons other than origins. The hierarchical cluster analysis was then undertaken to guide the predefined number of clusters, described as *K*, in *K*-means cluster analysis. This is a measure of dissimilarity, and one output is a dendrogram, which displays the rescaled distance between samples in terms of their isotope signatures. This exploratory analysis informs the *K* value by using the number of clusters on the dendrogram at different rescaled distance values. The hierarchical cluster analysis used the “between-groups average linkage” cluster method with a squared Euclidean distance interval measure.

## Supplementary Material

http://advances.sciencemag.org/cgi/content/full/5/3/eaau6078/DC1

Download PDF

## SUPPLEMENTARY MATERIALS

Supplementary material for this article is available at http://advances.sciencemag.org/cgi/content/full/5/3/eaau6078/DC1 Supplementary Materials and Methods Table S1. Element, side, and context for the sampled remains. Table S2. Enamel ^87^Sr/^86^Sr, δ^18^O, and δ^13^C isotope results with data on Sr concentration and carbonate replicates. Table S3. Results from sulfur (δ^34^S) isotope analysis. Table S4. Cluster membership based on a rescaled distance of two on the example dendrogram in fig. S1. Table S5. Characterization of the clusters in the *K* = 24 model, showing membership (*n*) at each site, isotope value ranges, and the maximum distance from the cluster center among members. Table S6. Results of *K* = 24 cluster analysis ordered by sample number, showing cluster membership for each sample and its distance from the cluster centre. Table S7. Results of the *K* = 24 cluster model, showing sample membership for each cluster. Table S8. Samples with highly radiogenic (>0.7131) ^87^Sr/^86^Sr values. Fig. S1. Example dendrogram output from the hierarchical cluster analysis in SPSS with annotated cluster numbers.
