# Supplementary material for: Multi-isotope analysis reveals that feasts in the Stonehenge environs and across Wessex drew people and animals from throughout Britain
Source: Sci Adv. 2019 Mar 13;5(3):eaau6078. doi: 10.1126/sciadv.aau6078 (PMC6415963; doi:10.1126/sciadv.aau6078)
Supplement: http://advances.sciencemag.org/cgi/content/full/5/3/eaau6078/DC1 [file supp_5_3_eaau6078__index.html]

Science Advances | Science Advances

## Supplementary Materials

**This PDF file includes:**

- Supplementary Materials and Methods
- Table S1. Element, side, and context for the sampled remains.
- Table S2. Enamel 87Sr/86Sr, δ18O, and δ13C isotope results with data on Sr concentration and carbonate replicates.
- Table S3. Results from sulfur (δ34S) isotope analysis.
- Table S4. Cluster membership based on a rescaled distance of two on the example dendrogram in fig. S1.
- Table S5. Characterization of the clusters in the *K* = 24 model, showing membership (*n*) at each site, isotope value ranges, and the maximum distance from the cluster center among members.
- Table S6. Results of *K* = 24 cluster analysis ordered by sample number, showing cluster membership for each sample and its distance from the cluster centre.
- Table S7. Results of the *K* = 24 cluster model, showing sample membership for each cluster.
- Table S8. Samples with highly radiogenic (>0.7131) 87Sr/86Sr values.
- Fig. S1. Example dendrogram output from the hierarchical cluster analysis in SPSS with annotated cluster numbers.

Download PDF

**Files in this Data Supplement:**

- Adobe PDF - aau6078\_SM.pdf
