# Supplementary material for: Multi-isotope analysis reveals that feasts in the Stonehenge environs and across Wessex drew people and animals from throughout Britain
Source: Sci Adv. 2019 Mar 13;5(3):eaau6078. doi: 10.1126/sciadv.aau6078 (PMC6415963; doi:10.1126/sciadv.aau6078)
Supplement: Download PDF [file aau6078_SM.pdf]

Science Advances
AAAS

advances.sciencemag.org/cgi/content/full/5/3/eaau6078/DC1

Supplementary Materials for

Multi-isotope analysis reveals that feasts in the Stonehenge environs and across Wessex drew people and animals from throughout Britain

R. Madgwick*, A. L. Lamb, H. Sloane, A. J. Nederbragt, U. Albarella, M. Parker Pearson, J. A. Evans

*Corresponding author. Email: madgwickrd3@cardiff.ac.uk

Published 13 March 2019, *Sci. Adv.* **5**, eaau6078 (2019)
DOI: 10.1126/sciadv.aau6078

This PDF file includes:

Supplementary Materials and Methods
Table S1. Element, side, and context for the sampled remains.
Table S2. Enamel $^{87}Sr/^{86}Sr$, $\delta^{18}O$, and $\delta^{13}C$ isotope results with data on Sr concentration and carbonate replicates.
Table S3. Results from sulfur ($\delta^{34}S$) isotope analysis.
Table S4. Cluster membership based on a rescaled distance of two on the example dendrogram in fig. S1.
Table S5. Characterization of the clusters in the $K = 24$ model, showing membership (n) at each site, isotope value ranges, and the maximum distance from the cluster center among members.
Table S6. Results of $K = 24$ cluster analysis ordered by sample number, showing cluster membership for each sample and its distance from the cluster centre.
Table S7. Results of the $K = 24$ cluster model, showing sample membership for each cluster.
Table S8. Samples with highly radiogenic (>0.7131) $^{87}Sr/^{86}Sr$ values.
Fig. S1. Example dendrogram output from the hierarchical cluster analysis in SPSS with annotated cluster numbers.

Supplementary Material

Supplementary Materials and Methods

Samples

Table S1. Element, side, and context for the sampled remains. WKPE has finds numbers listed rather than contexts. Contextual information was not available for Mount Pleasant, but all material was assigned to the Late Neolithic phase. When teeth are listed in the 'Collagen sample' field, bulk dentine was sampled, rather than bone.

Sample	Site	CTX/Find no.	Collagen sample	Enamel Sample	Side
DW01	Durrington Walls	593	Mandible	M1	L
DW02	Durrington Walls	593	Mandible	M1	R
DW03	Durrington Walls	593	Mandible	M1	R
DW04	Durrington Walls	593	Mandible	M1	L
DW05	Durrington Walls	593	Mandible	M1	R
DW06	Durrington Walls	593	Mandible	M1	R
DW07	Durrington Walls	593	Mandible	M1	L
DW08	Durrington Walls	593	Mandible	M1	R
DW09	Durrington Walls	593	Mandible	M1	L
DW10	Durrington Walls	593	Mandible	M1	R
DW11	Durrington Walls	593	Mandible	M1	R
DW12	Durrington Walls	593	Mandible	M1	R
DW13	Durrington Walls	593	Mandible	M1	R
DW14	Durrington Walls	593	Mandible	M1	L
DW15	Durrington Walls	593	Mandible	M1	L
DW16	Durrington Walls	593	Mandible	M1	R
DW17	Durrington Walls	593	Mandible	M1	R
DW18	Durrington Walls	593	Mandible	M1	L
DW19	Durrington Walls	593	Mandible	M1	L
DW20	Durrington Walls	593	Mandible	M1	L
DW21	Durrington Walls	593	Mandible	M1	R
DW22	Durrington Walls	593	Mandible	M1	L
DW23	Durrington Walls	593	Mandible	P4	R
DW24	Durrington Walls	593	Mandible	M1	R
DW25	Durrington Walls	593	Mandible	M1	L
DW26	Durrington Walls	593	Mandible	M1	L
DW27	Durrington Walls	867	Mandible	M1	L
DW28	Durrington Walls	867	Mandible	P3	R
DW29	Durrington Walls	867	Mandible	M1	R
DW30	Durrington Walls	867	Mandible	M1	L
DW31	Durrington Walls	856	Mandible	M1	R
DW32	Durrington Walls	637	Mandible	M1	L
DW33	Durrington Walls	637	Mandible	M1	R

DW34	Durrington Walls	593	Mandible	M1	L
DW35	Durrington Walls	724	Mandible	M1	R
DW36	Durrington Walls	724	Mandible	M1	L
DW37	Durrington Walls	586	Mandible	M1	R
DW38	Durrington Walls	586	Mandible	M1	L
DW39	Durrington Walls	586	Mandible	M1	R
DW40	Durrington Walls	586	Mandible	M1	R
DW41	Durrington Walls	786	Mandible	M1	R
DW42	Durrington Walls	1359	Mandible	M1	L
DW43	Durrington Walls	1369	Mandible	M1	R
DW44	Durrington Walls	1359	Mandible	M1	R
DW45	Durrington Walls	1359	Mandible	M1	L
DW46	Durrington Walls	1359	Mandible	M1	R
DW47	Durrington Walls	1381	Mandible	M1	L
DW48	Durrington Walls	1381	Mandible	M1	R
DW49	Durrington Walls	1381	Mandible	P4	R
DW50	Durrington Walls	1381	Mandible	M1	R
DW51	Durrington Walls	1381	Mandible	M1	R
DW52	Durrington Walls	1381	Mandible	M1	L
DW53	Durrington Walls	1381	Mandible	M1	L
DW54	Durrington Walls	547	Mandible	M1	L
DW55	Durrington Walls	547	Mandible	M1	R
DW56	Durrington Walls	652	Mandible	M1	R
DW57	Durrington Walls	652	Mandible	M1	R
DW58	Durrington Walls	652	Mandible	M1	L
DW59	Durrington Walls	652	Mandible	M1	R
DW60	Durrington Walls	652	Mandible	M1	R
DW61	Durrington Walls	652	Mandible	M1	R
DW62	Durrington Walls	652	Mandible	M1	L
DW63	Durrington Walls	652	Mandible	M1	R
DW64	Durrington Walls	652	Mandible	M1	L
DW65	Durrington Walls	652	Mandible	M1	R
DW66	Durrington Walls	641	Mandible	M1	L
DW67	Durrington Walls	641	Mandible	M1	R
DW68	Durrington Walls	608	Mandible	M1	L
DW69	Durrington Walls	608	Mandible	M1	L
DW70	Durrington Walls	608	Mandible	M1	L
DW71	Durrington Walls	641	Mandible	M1	L
DW72	Durrington Walls	641	Mandible	M1	R
DW73	Durrington Walls	641	Mandible	M1	L
DW74	Durrington Walls	641	Mandible	M1	R
DW75	Durrington Walls	641	Mandible	M1	R
DW76	Durrington Walls	641	Mandible	M1	L

DW77	Durrington Walls	641	Mandible	M1	R
DW78	Durrington Walls	1573	Mandible	M1	R
DW79	Durrington Walls	1406	Mandible	M1	L
DW80	Durrington Walls	1406	Mandible	M1	L
DW81	Durrington Walls	1406	Mandible	M1	R
DW82	Durrington Walls	1406	Mandible	M1	R
DW83	Durrington Walls	1394	Mandible	M1	L
DW84	Durrington Walls	1394	Mandible	M1	R
DW85	Durrington Walls	593	Mandible	M2	R
DW86	Durrington Walls	593	Mandible	M2	R
DW87	Durrington Walls	593	Mandible	M2	R
DW88	Durrington Walls	593	Mandible	M2	R
DW89	Durrington Walls	593	Mandible	M2	R
MP90	Mount Pleasant		Maxilla	M1	R
MP91	Mount Pleasant		Maxilla	M3	R
MP92	Mount Pleasant		Mandible	P4	R
MP93	Mount Pleasant		Maxilla	M1	R
MP94	Mount Pleasant		Maxilla	M1	R
MP95	Mount Pleasant		Maxilla	M2	R
MP96	Mount Pleasant		Maxilla	M1	L
MP97	Mount Pleasant		Maxilla	P3	R
MP98	Mount Pleasant		Maxilla	M3	R
MP99	Mount Pleasant		Mandible	M2	R
MP100	Mount Pleasant		Maxillary incisor	Incisor	L
MP101	Mount Pleasant		Mandibular premolar	P4	R
MP102	Mount Pleasant		Maxilla	P3	L
MP103	Mount Pleasant		Mandibular molar	M1	R
MP104	Mount Pleasant		Mandibular molar	M2	L
MP105	Mount Pleasant		Maxillary molar	M2	L
MP106	Mount Pleasant		Maxillary molar	M2	L
MP107	Mount Pleasant		Mandibular molar	M1	R
WK108	WKPE	50836	Mandible	M1	L
WK109	WKPE	9547	Mandible	M1	L
WK110	WKPE	50417	Mandible	M1	R
WK111	WKPE	50990	Mandible	M1	L
WK112	WKPE	2321	Mandible	M1	R
WK113	WKPE	2414	Maxilla	M1	R
WK114	WKPE	2301	Maxilla	M1	R
WK115	WKPE	2222	Maxilla	M1	R
WK116	WKPE	2278	Maxilla	M1	L
WK117	WKPE	2048	Maxilla	M1	R
WK118	WKPE	2148	Maxilla	M1	R
WK119	WKPE	2353	Maxilla	M1	L

WK120	WKPE	6032	Mandible	M1	R
WK121	WKPE	50857	Mandible	M1	R
WK122	WKPE	41041	Mandible	M1	L
WK123	WKPE	1025	Mandible	M1	L
MD124	Marden	16	Mandible	Incisor	L
MD125	Marden	16	Mandible	M1	L
MD126	Marden	16	Maxilla	M1	L
MD127	Marden	16	Maxilla	M3	R
MD128	Marden	16	Mandibular incisor	Incisor	L
MD129	Marden	16	Mandible	M1	L
MD130	Marden	25	Mandible	M3	L
MD131	Marden	25	Mandible	M1	L

$^{87}Sr/^{86}Sr$ isotope analysis

Dental enamel was sampled from all individuals for $^{87}Sr/^{86}Sr$ isotope analysis. Only teeth that were in wear were selected to ensure that they had neared completion of the mineralisation process and were therefore resistant to diagenetic alteration. Wherever possible enamel slices were sampled from 2mm away from the enamel-root junction (ERJ) of lower first molars to provide comparable signals of early life origins (c. 2-6 months). In addition, the buccal surface of the distal cusp unit was targeted when it was sufficiently well preserved to be sampled. In some instances other M1 cusps or other teeth were analysed due to sample availability, but efforts were made to analyse areas of enamel that developed at a similar time and all specimens pertained to approximately the first eight months of life (see Table S1).

$\delta^{18}O$ isotope analysis

Every effort was made to target enamel that developed and mineralised around the same age across all samples (crown complete around 2-3 months, mineralisation estimated to be complete around 6 months). For lower M1s, which constituted the vast majority of the samples (Table S1), a strip of enamel approximately 2-3mm in breadth was sampled from 2mm from the root-enamel junction on the lingual surface of the distal cusp unit. When the lingual surface or the distal cusp unit were damaged, other portions of the tooth were sampled that would have developed at approximately the same time. Similarly, when other dental specimens were analysed sampling targeted portions of the tooth that would have developed around the same time of life. This was not possible for all samples and enamel from the M3s (N=4) and P4s (N=4) would have developed between the age of 5 and 12 months and mineralise slightly after this period.

As $\delta^{18}O$ isotope values principally relate to climate (see Fig. 4), varied seasonal timing of the development of sampled enamel could affect the comparability of results. It was for this reason that efforts were made to sample enamel that developed at a similar age. This assumes a single birthing season in the spring, which, although not empirically demonstrated, is by far the mostly likely scenario for Late Neolithic pig husbandry (*2*). In any case the effects of seasonal climatic variation on values is limited in pig molars (*38*) and is certain to be particularly limited in first molars, which develop rapidly. The sampling of enamel that developed at a slightly later stage

should not therefore have a substantial impact on values. Variation in canines is far larger (*38*), but none have been analysed in this study. $\delta^{18}O$ isotope values can also be affected by a nursing signal, prior to infants being weaned and also by dietary variation. It cannot be discounted that these variables could have impacted on isotope signals in some instances. The weaning age for pigs is variable and it has not been studied in detail for pigs in prehistoric Britain. Pigs can be weaned from an age of ten days up to four months. Organised farming of pigs has tended to encourage weaning at around 30 days of age but this can extend to 5 weeks for naturally farmed pig. Weaning ages of 4 months are rare in farming systems and even in free range strategies employed in Sardinia, 3 months have been shown to be the maximum with one month being more typical. In modern pigs the sow's milk dries off between three and eight weeks and therefore the input of a nursing signal would be strongest in the first three weeks. M1 crown development completes at 3 months and final mineralisation should be complete around 6 months. Enamel was sampled from near the REJ and therefore would have developed slightly before 3 months of age and fully mineralised later. Therefore it is possible that some nursing signal could be present in late weaned animals and values will not always represent a climatic signal relating entirely to ingested drinking water. However, this effect is likely to be rare and slight and the signal would be dampened by the completion of mineralisation after crown development. The normal distribution of the data also supports the negligible impact of a nursing signal, as this would result in some skewing (Shapiro Wilk 0.992, sig. 0.675).

$\delta^{13}C$, $\delta^{15}N$ and $\delta^{34}S$ isotope analysis

Collagen from bone or dentine was sampled from all individuals for $\delta^{13}C$, $\delta^{15}N$ and $\delta^{34}S$ isotope analysis. Wherever possible compact bone from the mandibular body or ramus (N=105) was analysed but in some instances it was necessary to sample maxillary bone (N=18) or dentine (N=8), see Table S1. Dentine does not remodel after production and therefore provides a signature for a specific period in the animal’s development. However, relatively large sections of root extending from the apex (i.e. the latest forming part) were sampled and will therefore provide an averaged signal, rather than a short-term snapshot. Bone constantly remodels and therefore provides an averaged signature for the years before death, which is likely to cover the whole duration of the pigs’ lives.

Isotope ratios of carbon, nitrogen and sulphur were measured by continuous flow-elemental analysis-isotope ratio mass spectrometry (CF-EA-IRMS). All sulphur isotope analysis was undertaken at the NERC Isotope Geosciences Laboratory facility at Keyworth in Nottinghamshire. $\delta^{13}C$ and $\delta^{15}N$ isotope analysis was undertaken at the School of Earth and Ocean Sciences, Cardiff University (N=78), the NERC Isotope Geosciences Laboratory facility at Keyworth (N=32) and the McDonald Institute for Archaeological Research, University of Cambridge (N=21). At Cardiff University a Flash 1112 series elemental analyser coupled to a Thermo Delta V Advantage was used in analysis. At the NERC Isotope Geosciences Laboratory facility, the instrumentation comprises an elemental analyser (Flash/EA) coupled to a ThermoFinnigan Delta Plus XL isotope ratio mass spectrometer via a ConFlo III interface. At the McDonald Institute an automated elemental analyser coupled in continuous-flow mode to an isotope-ratio monitoring mass spectrometer (Costech elemental analyser coupled to a Finnigan MAT253 mass spectrometer) was used. Results are presented in Table S3.

Analysis of all samples complied with collagen quality control criteria for sulphur (C:S ratio of 600±300; N:S ratio of 200±100, see Table S3). The mean 1σ reproducibility for mass spectrometry controls (powdered gelatine standard [M1360p] from British Drug Houses) in the 13 runs used in the $\delta^{34}S$ dataset was ±0.20‰. A total of 123 sample replicates provided an average standard deviation of 0.36. This is acceptable, particularly considering the wide range of values produced in $\delta^{34}S$ isotope analysis and the conservative approach to their interpretation in this study. There is very little sulphur in mammalian collagen (<0.35%) and values can range from -20‰ to +19‰ depending on diet and environment (*19*). Therefore greater duplicate variability must be expected than for $\delta^{13}C$ and $\delta^{15}N$ values. For $\delta^{13}C$ and $\delta^{15}N$ isotopes, replicate analysis of well-mixed samples provided average standard deviations of 0.090 for $\delta^{13}C$ and 0.087 for $\delta^{15}N$. In house standards supported the reliability of analyses[1]. All samples had a C:N ratio of 3.2 (±0.3) and were therefore considered to be sufficiently well preserved to yield reliable $\delta^{13}C$ and $\delta^{15}N$ values.

Results

$^{87}Sr/^{86}Sr$ and $\delta^{18}O$ isotope analysis (Table S2)

Table S2. Enamel $^{87}Sr/^{86}Sr$, $\delta^{18}O$, and $\delta^{13}C$ isotope results with data on Sr concentration and carbonate replicates.

Sample	Site	Sr ppm	$^{87}Sr/^{86}Sr$	$\delta^{18}O$ VSMOW	$\delta^{13}C$ VPDB	Replicates
DW01	Durrington Walls	123	0.708709	25.0	-14.3	2
DW02	Durrington Walls	154	0.709497	25.6	-12.8	1
DW03	Durrington Walls	127	0.709113	25.5	-14.7	1
DW04	Durrington Walls	144	0.710039	26.6	-14.3	2
DW05	Durrington Walls	158	0.711883	24.0	-13.9	1
DW06	Durrington Walls	125	0.709323	25.4	-13.2	1
DW07	Durrington Walls	75	0.709702	27.7	-12.7	1
DW08	Durrington Walls	93	0.708646	25.0	-14.5	1
DW09	Durrington Walls	102	0.708498	25.8	-14.3	1
DW10	Durrington Walls	197	0.710711	26.7	-14.3	1
DW11	Durrington Walls	130	0.712633	25.4	-13.6	1
DW12	Durrington Walls	110	0.710325	25.2	-14.4	2
DW13	Durrington Walls	90	0.709026	25.9	-14.6	1
DW14	Durrington Walls	119	0.712339	25.4	-14.3	1
DW15	Durrington Walls	217	0.709432	26.1	-14.3	1
DW16	Durrington Walls	120	0.708323	27.0	-13.0	1
DW17	Durrington Walls	149	0.710555	25.0	-13.6	1

[1] Laboratory Standards comprised supermarket gelatine and caffeine at Cardiff, a powdered gelatine standard (M1360p) from British Drug Houses and a modern cow at NIGL and caffeine, USGS40, nylon, alanine and a bovine liver standard at Cambridge.

DW18	Durrington Walls	141	0.708465	25.4	-13.6	2
DW19	Durrington Walls	104	0.708767	27.0	-14.7	1
DW20	Durrington Walls	189	0.710734	25.2	-14.9	1
DW21	Durrington Walls	149	0.708498	25.3	-13.8	1
DW22	Durrington Walls	124	0.709173	26.2	-14.6	2
DW23	Durrington Walls	90	0.708261	26.6	-14.9	1
DW24	Durrington Walls	196	0.710557	23.6	-13.5	2
DW25	Durrington Walls	93	0.708330	27.3	-14.6	1
DW26	Durrington Walls	125	0.709503	26.2	-14.6	1
DW27	Durrington Walls	80	0.710133	25.2	-14.1	1
DW28	Durrington Walls	117	0.711130	26.3	-14.7	1
DW29	Durrington Walls	84	0.708566	27.4	-14.3	2
DW30	Durrington Walls	82	0.707955	26.0	-14.9	1
DW31	Durrington Walls	169	0.717206	25.2	-14.7	1
DW32	Durrington Walls	102	0.709371	26.6	-15.0	1
DW33	Durrington Walls	151	0.708554	26.4	-14.2	1
DW34	Durrington Walls	134	0.709147	26.0	-14.3	1
DW35	Durrington Walls	97	0.710316	25.9	-14.4	1
DW36	Durrington Walls	83	0.709561	26.2	-14.6	1
DW37	Durrington Walls	153	0.709754	26.4	-14.3	1
DW38	Durrington Walls	163	0.712700	24.4	-14.6	1
DW39	Durrington Walls	99	0.710221	24.8	-14.1	2
DW40	Durrington Walls	137	0.714796	26.5	-14.5	1
DW41	Durrington Walls	138	0.708640	26.2	-14.9	1
DW42	Durrington Walls	110	0.709412	27.7	-14.8	1
DW43	Durrington Walls	215	0.709390	26.1	-14.4	1
DW44	Durrington Walls	78	0.709692	27.0	-13.5	1
DW45	Durrington Walls	142	0.711295	27.3	-14.0	1
DW46	Durrington Walls	140	0.709412	26.3	-14.8	1
DW47	Durrington Walls	128	0.708387	25.8	-14.6	1
DW48	Durrington Walls	131	0.708016	26.8	-14.4	1
DW49	Durrington Walls	195	0.709548	25.4	-14.6	1
DW50	Durrington Walls	98	0.708537	26.9	-12.5	1
DW51	Durrington Walls	150	0.708521	26.5	-14.8	1
DW52	Durrington Walls	151	0.709539	26.8	-14.4	1
DW53	Durrington Walls	138	0.708464	26.1	-14.9	1
DW54	Durrington Walls	83	0.709009	25.7	-14.4	2
DW55	Durrington Walls	207	0.712693	23.7	-13.9	2
DW56	Durrington Walls	167	0.711637	26.7	-14.9	2
DW57	Durrington Walls	74	0.708465	25.6	-13.9	1
DW58	Durrington Walls	313	0.711613	25.3	-12.2	1

DW59	Durrington Walls	80	0.709858	27.5	-14.9	1
DW60	Durrington Walls	140	0.714861	25.2	-14.8	1
DW61	Durrington Walls	182	0.708961	25.6	-14.4	1
DW62	Durrington Walls	98	0.709722	25.1	-14.7	1
DW63	Durrington Walls	61	0.709389	27.5	-14.8	1
DW64	Durrington Walls	134	0.708384	25.7	-14.7	1
DW65	Durrington Walls	100	0.709158	26.2	-15.0	1
DW66	Durrington Walls	70	0.708240	26.1	-13.6	1
DW67	Durrington Walls	822	0.710096	25.0	-13.7	1
DW68	Durrington Walls	121	0.709223	24.8	-13.2	1
DW69	Durrington Walls	128	0.710246	25.8	-14.1	1
DW70	Durrington Walls	176	0.709379	23.7	-12.5	1
DW71	Durrington Walls	92	0.709165	24.7	-13.6	2
DW72	Durrington Walls	123	0.709420	25.3	-13.0	2
DW73	Durrington Walls	140	0.709668	25.3	-13.7	2
DW74	Durrington Walls	165	0.708935	24.6	-14.0	2
DW75	Durrington Walls	160	0.709377	25.6	-13.7	1
DW76	Durrington Walls	183	0.709563	26.1	-14.0	1
DW77	Durrington Walls	183	0.711141	24.3	-11.4	2
DW78	Durrington Walls	137	0.708961	26.7	-14.9	1
DW79	Durrington Walls	115	0.709071	24.8	-13.1	2
DW80	Durrington Walls	215	0.711479	25.9	-14.6	1
DW81	Durrington Walls	167	0.710098	26.5	-14.4	2
DW82	Durrington Walls	114	0.708760	26.1	-14.6	2
DW83	Durrington Walls	219	0.708847	26.9	-14.1	2
DW84	Durrington Walls	129	0.708118	26.0	-14.5	2
DW85	Durrington Walls	107	0.708031	27.2	-14.3	1
DW86	Durrington Walls	111	0.709349	26.6	-14.3	1
DW87	Durrington Walls	135	0.709324	26.6	-15.0	1
DW88	Durrington Walls	268	0.710338	25.8	-14.1	1
DW89	Durrington Walls	153	0.710116	24.4	-12.9	2
MP90	Mount Pleasant	304	0.709114	26.5	-13.3	1
MP91	Mount Pleasant	169	0.710223	26.3	-13.3	1
MP92	Mount Pleasant	114	0.708787	24.1	-14.6	2
MP93	Mount Pleasant	168	0.708291	25.7	-14.2	2
MP94	Mount Pleasant	179	0.711146	24.1	-14.4	1
MP95	Mount Pleasant	166	0.711280	24.7	-14.1	2
MP96	Mount Pleasant	102	0.709641	23.2	-12.9	2
MP97	Mount Pleasant	178	0.711133	23.9	13.8	2
MP98	Mount Pleasant	169	0.710740	23.7	-15.0	1
MP99	Mount Pleasant	158	0.709759	25.2	-13.0	1

MP100	Mount Pleasant	167	0.710668	24.4	-12.7	2
MP101	Mount Pleasant	205	0.710031	25.6	-12.2	1
MP102	Mount Pleasant	148	0.709707	24.5	-13.8	1
MP103	Mount Pleasant	156	0.710410	24.6	-12.7	1
MP104	Mount Pleasant	78	0.708420	23.7	-12.9	1
MP105	Mount Pleasant	84	0.708484	25.7	-12.9	1
MP106	Mount Pleasant	124	0.708472	25.6	-13.2	2
MP107	Mount Pleasant	284	0.709545	28.9	-13.0	2
WK108	West Kennet PE	85	0.708170	27.6	-14.7	2
WK109	West Kennet PE	183	0.711857	24.6	-14.3	1
WK110	West Kennet PE	105	0.709252	25.9	-14.0	1
WK111	West Kennet PE	115	0.709531	26.4	-14.4	1
WK112	West Kennet PE	103	0.708854	25.8	-14.7	2
WK113	West Kennet PE	149	0.711667	25.0	-14.3	1
WK114	West Kennet PE	168	0.710597	25.6	-13.7	1
WK115	West Kennet PE	132	0.709376	25.8	-15.1	1
WK116	West Kennet PE	123	0.708747	27.2	-14.4	1
WK117	West Kennet PE	181	0.708965	26.2	-14.9	1
WK118	West Kennet PE	115	0.713141	24.4	-13.2	1
WK119	West Kennet PE	263	0.711384	24.8	-14.4	1
WK120	West Kennet PE	115	0.708486	26.4	-15.2	1
WK121	West Kennet PE	215	0.713262	25.1	-15.4	2
WK122	West Kennet PE	227	0.710136	25.1	-13.0	1
WK123	West Kennet PE	98	0.708246	25.5	-15.0	2
MD124	Marden	243	0.710377	23.2	-13.6	2
MD125	Marden	133	0.710415	26.4	-13.0	1
MD126	Marden	156	0.716593	25.9	-13.4	2
MD127	Marden	13	0.709808	24.4	-14.0	2
MD128	Marden	155	0.713471	25.8	-13.8	2
MD129	Marden	177	0.714133	23.88	-13.0	1
MD130	Marden	194	0.711624	24.0	-12.9	2
MD131	Marden	96	0.708612	27.4	-14.2	1

$\delta^{34}S$, $\delta^{13}C$ and $\delta^{15}N$ isotope analysis (Table S3)

Table S3. Results from sulfur ($\delta^{34}S$) isotope analysis. The number of replicates pertains to the successful analyses that have been used in calculations. Samples with poor replicate results (N=8) have been omitted. N:S and C:S fields show atomic ratios.

Sample	Site	$\delta^{34}S$	%S	N:S	C:S	Reps	$\delta^{13}C$	$\delta^{15}N$	%C	%N	C:N	Reps
DW01	Durrington Walls	15.1	0.15	180	548	2	-21.3	6.7	31.6	12.1	3.1	3
DW02	Durrington Walls	14.0	0.15	174	573	2	-20.9	6.7	32.5	11.5	3.3	3
DW03	Durrington Walls	11.9	0.14	172	572	2	-20.8	6.6	30.5	10.7	3.3	3
DW04	Durrington Walls	14.3	0.13	184	594	2	-20.5	6.4	27.9	10.1	3.2	3
DW05	Durrington Walls	10.9	0.22	131	416	2	-20.5	7.0	34.1	12.6	3.2	2
DW06	Durrington Walls	8.0	0.15	160	494	2	-20.0	6.3	28.1	10.6	3.1	3
DW07	Durrington Walls	10.0	0.14	202	662	2	-20.6	7.0	35.9	12.8	3.3	3
DW08	Durrington Walls	13.8	0.19	136	454	2	-19.8	6.3	32.3	11.2	3.4	3
DW09	Durrington Walls	14.3	0.17	148	468	2	-20.9	5.9	30.4	11.2	3.2	3
DW10	Durrington Walls	7.1	0.17	168	570	2	-20.9	7.5	35.8	12.3	3.4	2
DW11	Durrington Walls	11.7	0.13	181	605	2	-20.3	6.8	30.5	10.6	3.4	2
DW12	Durrington Walls	14.6	0.16	182	585	2	-20.3	6.3	35.8	13.0	3.2	3
DW13	Durrington Walls	14.0	0.12	177	528	2	-20.0	6.0	23.5	9.2	3.0	3
DW14	Durrington Walls	16.1	0.14	176	517	2	-20.6	5.2	26.6	10.6	2.9	3
DW15	Durrington Walls	4.0	0.15	160	509	2	-21.1	6.2	28.6	10.5	3.2	3
DW16	Durrington Walls	5.8	0.16	187	625	2	-20.3	6.2	36.6	12.8	3.3	2
DW17	Durrington Walls	15	0.18	175	528	2	-20.3	6.6	36.1	14.0	3.0	3
DW18	Durrington Walls	3.5	0.11	185	550	2	-19.9	7.1	22.2	8.7	3.0	3
DW19	Durrington Walls	15.4	0.12	187	627	2	-20.0	7.1	29.3	10.2	3.4	2
DW20	Durrington Walls	7.0	0.18	174	585	2	-20.9	7.4	40.2	14.0	3.4	2
DW21	Durrington Walls	3.4	0.14	160	480	2	-19.9	7.4	25.8	10.1	3.0	3
DW22	Durrington Walls	8.1	0.17	170	574	2	-21.9	5.9	36.1	12.5	3.4	2
DW23	Durrington Walls	12.3	0.18	157	472	2	-21.0	6.9	31.8	12.4	3.0	3
DW24	Durrington Walls	10.8	0.14	205	573	2	-21.3	7.5	31.1	12.5	2.9	3
DW25	Durrington Walls	14.2	0.17	167	564	2	-19.7	7.9	36.1	12.5	3.4	2
DW26	Durrington Walls	9.2	0.19	166	476	2	-19.8	7.1	34.2	13.9	2.9	3
DW27	Durrington Walls	11.9	0.11	183	614	2	-20.1	6.3	26.4	9.2	3.4	2
DW28	Durrington Walls	12.8	0.19	152	450	2	-20.9	5.8	31.5	12.4	3.0	3
DW29	Durrington Walls	15.3	0.15	178	595	2	-20.3	7.5	32.4	11.3	3.4	2
DW30	Durrington Walls	15.2	0.16	178	593	2	-20.2	5.8	36.4	12.7	3.3	2
DW31	Durrington Walls	11.6	0.14	161	547	2	-21.0	6.5	28.6	9.8	3.4	2
DW32	Durrington Walls	11.7	0.13	178	593	2	-20.6	7.2	28.5	10.0	3.3	2
DW33	Durrington Walls	13.6	0.17	178	603	2	-21.1	7.0	38.0	13.1	3.4	2
DW34	Durrington Walls	13.7	0.14	186	631	2	-21.7	6.9	32.0	11.0	3.4	2
DW35	Durrington Walls	12.6	0.20	148	504	2	-20.5	5.4	37.7	12.9	3.4	2

DW36	Durrington Walls	13.7	0.14	164	554	2	-20.3	5.3	29.4	10.1	3.4	2
DW37	Durrington Walls	7.7	0.16	159	544	2	-20.9	7.5	33.5	11.4	3.4	2
DW38	Durrington Walls	16.4	0.13	190	637	2	-20.3	6.1	31.6	11.0	3.4	2
DW39	Durrington Walls	13.7	0.14	172	582	2	-20.7	6.1	31.5	10.8	3.4	2
DW40	Durrington Walls	14.2	0.14	187	623	3	-21.2	6.6	32.9	11.5	3.3	2
DW41	Durrington Walls	14.1	0.17	175	593	2	-21.4	6.6	37.9	13.1	3.4	2
DW42	Durrington Walls	10.6	0.18	165	556	2	-21.7	6.9	36.9	12.8	3.4	2
DW43	Durrington Walls	2.9	0.12	213	711	2	-21.0	6.5	31.4	11.0	3.3	2
DW44	Durrington Walls	13.9	0.14	165	571	2	-21.5	6.1	29.3	9.9	3.5	2
DW45	Durrington Walls	11.7	0.11	167	565	2	-20.9	6.6	23.2	8.0	3.4	2
DW46	Durrington Walls	8.3	0.12	173	589	2	-21.1	6.3	27.0	9.2	3.4	2
DW47	Durrington Walls	5.5	0.13	168	579	2	-20.7	6.5	28.3	9.6	3.5	2
DW48	Durrington Walls	7.7	0.14	182	611	2	-20.7	6.7	32.0	11.1	3.4	2
DW49	Durrington Walls	11.9	0.12	193	642	2	-21.7	6.4	29.0	10.2	3.3	3
DW50	Durrington Walls	11.4	0.14	187	548	2	-21.4	6.6	27.8	11.1	2.9	3
DW51	Durrington Walls	8.7	0.14	134	458	2	-20.0	7.0	24.0	8.2	3.4	2
DW52	Durrington Walls	13.7	0.12	205	694	2	-20.9	7.6	31.7	10.9	3.4	2
DW53	Durrington Walls	10.5	0.18	159	539	2	-21.4	7.2	36.3	12.5	3.4	2
DW54	Durrington Walls	13.0	0.10	167	510	2	-20.8	7.3	19.5	7.4	3.1	2
DW55	Durrington Walls	-	-	-	-	-	-21.1	6.9	24.4	9.3	3.1	2
DW56	Durrington Walls	12.7	0.13	205	615	2	-21.5	6.4	29.9	11.6	3.0	2
DW57	Durrington Walls	14.8	0.08	195	594	2	-20.1	6.0	16.9	6.5	3.1	2
DW58	Durrington Walls	14.2	0.11	181	553	2	-20.5	7.3	23.8	9.1	3.1	2
DW59	Durrington Walls	12.7	0.09	185	564	2	-20.6	8.0	18.1	6.9	3.1	2
DW60	Durrington Walls	12.8	0.11	164	508	2	-21.1	5.8	21.7	8.2	3.1	2
DW61	Durrington Walls	14.4	0.13	162	509	2	-20.8	6.0	24.5	9.1	3.2	2
DW62	Durrington Walls	10.8	0.13	154	471	2	-20.8	5.9	22.8	8.7	3.1	2
DW63	Durrington Walls	12.2	0.14	138	429	2	-20.6	6.8	22.2	8.3	3.1	2
DW64	Durrington Walls	9.1	0.16	165	508	2	-20.9	6.8	29.7	11.3	3.1	2
DW65	Durrington Walls	7.9	0.13	179	546	2	-21.1	6.7	26.0	9.9	3.1	2
DW66	Durrington Walls	-	-	-	-	-	-20.4	7.0	21.4	8.1	3.1	2
DW67	Durrington Walls	12.9	0.09	135	420	2	-21.2	5.6	13.8	5.2	3.1	3
DW68	Durrington Walls	8.9	0.09	186	574	2	-20.7	6.9	19.3	7.3	3.1	2
DW69	Durrington Walls	13.7	0.11	160	496	2	-20.2	5.5	19.8	7.5	3.1	3
DW70	Durrington Walls	5.9	0.14	187	562	2	-20.1	7.5	30.2	11.7	3.0	2
DW71	Durrington Walls	6.6	0.16	166	502	2	-20.3	7.7	29.5	11.4	3.0	2
DW72	Durrington Walls	5.0	0.14	133	413	2	-20.5	5.2	22.3	8.4	3.1	2
DW73	Durrington Walls	-1.6	0.15	164	501	2	-20.3	5.7	27.9	10.6	3.1	2
DW74	Durrington Walls	3.7	0.12	181	555	2	-20.9	7.8	25.6	9.8	3.1	2
DW75	Durrington Walls	16.3	0.09	151	467	2	-21.2	5.8	15.3	5.8	3.1	2
DW76	Durrington Walls	-	-	-	-	-	-20.5	5.5	25.7	9.8	3.1	3

DW77	Durrington Walls	8.6	0.13	175	536	2	-20.1	6.8	26.9	10.3	3.1	2
DW78	Durrington Walls	13.4	0.18	183	569	2	-21.2	6.3	38.9	14.6	3.1	2
DW79	Durrington Walls	4.5	0.15	140	436	2	-21.7	6.3	23.9	9.0	3.1	2
DW80	Durrington Walls	13.1	0.11	209	646	2	-20.4	7.2	26.3	9.9	3.1	2
DW81	Durrington Walls	11.6	0.13	159	492	2	-19.9	6.4	24.8	9.3	3.1	3
DW82	Durrington Walls	10.2	0.13	174	540	2	-21.6	6.1	25.6	9.6	3.1	2
DW83	Durrington Walls	6.5	0.14	166	524	2	-21	6.6	27.0	10.0	3.2	2
DW84	Durrington Walls	15.8	0.14	184	561	2	-21.1	6.4	29.2	11.2	3.1	2
DW85	Durrington Walls	19.6	0.07	189	576	2	-20.7	6.6	15.2	5.8	3.0	3
DW86	Durrington Walls	14.5	0.15	171	533	2	-21.1	6.7	30.7	11.5	3.1	2
DW87	Durrington Walls	16.2	0.09	195	606	2	-21.2	6.8	19.8	7.4	3.1	2
DW88	Durrington Walls	12.8	0.17	172	527	2	-20.5	6.9	32.8	12.5	3.1	2
DW89	Durrington Walls	5.7	0.15	197	597	2	-20.4	6.6	32.7	12.6	3.0	2
MP90	Mount Pleasant	15.5	0.13	194	601	2	-21.6	7.5	29.3	11.0	3.1	2
MP91	Mount Pleasant	14.9	0.20	174	541	2	-21.8	5.8	39.8	15.0	3.1	2
MP92	Mount Pleasant	14.1	0.19	179	550	2	-20.9	6.8	39.3	14.9	3.1	2
MP93	Mount Pleasant	15.7	0.21	158	494	2	-21.3	6.9	38.9	14.5	3.1	2
MP94	Mount Pleasant	15.9	0.16	201	564	2	-21.5	6.0	33.4	11.8	3.3	2
MP95	Mount Pleasant	14.0	0.20	166	515	2	-21.7	6.5	38.1	14.3	3.1	2
MP96	Mount Pleasant	2.8	0.16	185	586	2	-21.2	6.8	35	12.9	3.2	2
MP97	Mount Pleasant	17.9	0.10	187	590	2	-21.5	6.1	23.1	8.5	3.2	2
MP98	Mount Pleasant	-	-	-	-	-	-21.9	6.4	35.9	13.2	3.2	2
MP99	Mount Pleasant	16.2	0.08	165	526	2	-20.9	4.5	16.5	6.0	3.2	3
MP100	Mount Pleasant	-	-	-	-	-	-21.5	7.1	32.0	11.9	3.1	2
MP101	Mount Pleasant	14.1	0.10	193	592	2	-21.9	6.8	22.7	8.7	3.1	2
MP102	Mount Pleasant	5.8	0.11	186	572	2	-21.6	7.1	22.6	8.6	3.1	3
MP103	Mount Pleasant	18.2	0.11	153	470	2	-21.2	6.3	20.1	7.6	3.1	3
MP104	Mount Pleasant	11.8	0.10	143	444	3	-22.4	6.8	16.3	6.1	3.1	2
MP105	Mount Pleasant	16.1	0.10	171	536	2	-21.9	6.0	20.1	7.5	3.1	2
MP106	Mount Pleasant	18.8	0.09	167	529	2	-23.1	6.2	17.0	6.3	3.2	2
MP107	Mount Pleasant	14.2	0.09	225	685	2	-21.4	9.1	22.7	8.7	3	2
WK108	West Kennet PE	14.5	0.12	189	580	3	-22.3	6.4	25	9.5	3.1	2
WK109	West Kennet PE	-	-	-	-	-	-21.3	5.9	26.1	9.3	3.3	2
WK110	West Kennet PE	14.6	0.11	219	677	2	-21.6	6.0	28.7	10.8	3.1	2
WK111	West Kennet PE	13.7	0.14	182	564	2	-20.6	6.0	30.3	11.4	3.1	2
WK112	West Kennet PE	9.1	0.06	186	599	2	-21.2	6.8	13.2	4.9	3.2	2
WK113	West Kennet PE	15.7	0.08	155	510	2	-22.4	5.1	14.7	5.2	3.2	2
WK114	West Kennet PE	6.9	0.10	124	419	2	-21.0	6.3	15.9	5.5	3.4	2
WK115	West Kennet PE	14.8	0.08	171	541	2	-21.4	6.0	16.7	6.2	3.2	2
WK116	West Kennet PE	12.6	0.11	127	413	2	-22.3	6.6	17.0	6.1	3.3	2
WK117	West Kennet PE	9.4	0.13	189	578	2	-21.2	5.5	27.1	10.3	3.1	2

WK118	West Kennet PE	8.4	0.06	142	455	2	-21.2	7.1	10.8	3.9	3.2	3
WK119	West Kennet PE	17.5	0.11	173	542	1	-21.5	5.6	22.5	8.4	3.1	2
WK120	West Kennet PE	15.1	0.11	163	507	3	-21.6	3.7	21.3	8.0	3.1	1
WK121	West Kennet PE	15.6	0.11	151	464	2	-20.8	5.8	18.5	7.0	3.1	2
WK122	West Kennet PE	15.7	0.11	149	465	2	-20.7	3.4	18.6	6.9	3.1	2
WK123	West Kennet PE	16.2	0.08	189	584	2	-21.6	4.9	18.0	6.8	3.1	2
MD124	Marden	7.0	0.06	128	394	2	-21.6	5.9	8.6	3.3	3.1	2
MD125	Marden	-	-	-	-	-	-20.3	5.9	10.7	4.0	3.1	2
MD126	Marden	11.5	0.08	208	635	2	-21.3	6.2	19.9	7.6	3.1	2
MD127	Marden	-	-	-	-	-	-21.5	5.1	11.3	4.3	3.1	2
MD128	Marden	10.7	0.09	163	492	2	-21.9	6.8	16.9	6.5	3.0	2
MD129	Marden	6.5	0.06	196	597	2	-21.2	6.5	13.4	5.1	3.1	2
MD130	Marden	7.4	0.06	169	492	2	-21.1	6.6	10.6	4.2	2.9	2
MD131	Marden	13.0	0.07	131	406	2	-22.0	5.0	10.0	3.8	3.1	3

The range of $\delta^{18}O$ values in the dataset is the same as the full range for meteoric water from the British Isles, with the exception of an outlying sample from Mount Pleasant (MP 107, 28.9‰). This sample has $^{87}Sr/^{86}Sr$ and $\delta^{34}S$ isotope values characteristic of the local environs but is a clear outlier in terms of $\delta^{15}N$ (9.1‰), which was analysed on a molar root. This high value means that the $\delta^{18}O$ can be explained by this animal having an unusual diet and therefore it can be excluded from consideration in defining the local $\delta^{18}O$ range. Evidence indicates that this sample was anomalous and that unusual diets or weaning effects did not have a substantial impact on the dataset as there is no correlation between the $\delta^{18}O$ and $\delta^{15}N$ values (Pearson 0.151, sig. 0.85) and the dataset is normally distributed (Shapiro Wilk 0.992, sig. 0.675). Skewness would be expected if diet and nursing had a substantial impact on the data. The rest of the dataset has the same range as meteoric water from the British Isles. It would be expected for drinking water values to have a slightly greater range than enamel in pigs (*32*) and therefore the range in the pig dataset is likely to equate to the full UK spectrum.

Cluster Analysis

Cluster analysis was employed in order to group samples with similar isotope signals using the three provenancing indices ($^{87}Sr/^{86}Sr$, $\delta^{34}S$, $\delta^{18}O$). This analysis has the potential to indicate the degree of diversity in origins. The first step in analysis was hierarchical clustering. Hierarchical cluster analysis builds a hierarchy of clusters, which effectively demonstrates the proximity of cases (in this instance pigs) in terms of a range of variables (in this instance provenancing isotope data). The dissimilarity of samples is assessed using the Euclidean distance interval measure. This uses the square root of the sum of the squared differences between values for the samples and is commonly used for interval data. This initial stage of analysis informed approaches taken (in terms of the number of defined clusters) in the next stage: K-means cluster analysis. K-means cluster analysis groups cases into a pre-defined number of clusters, with each case assigned to the cluster that has the closest mean values. Multiple K-means cluster models can be run and intra-cluster isotope variability must be scrutinised to assess whether the model is archaeologically

valid (i.e. whether clusters are sufficiently homogenous to represent distinct regions or whether they are too heterogeneous and must therefore comprise more than one region). If intra-cluster ranges are too diverse, a new model with a larger pre-defined numbers of clusters can be run. On the example dendrogram in fig. S1, the red arrow indicates a rescaled distance of two. Using the rescaled distance of two to generate a K value provides eight clusters in this instance. These are numbered in fig. S1 and cases are listed by cluster for clarity in Table S4. If a rescaled distance of three was chosen to generate a K value, then six clusters would be provided, as clusters 2/3 and 5/6 would be combined.

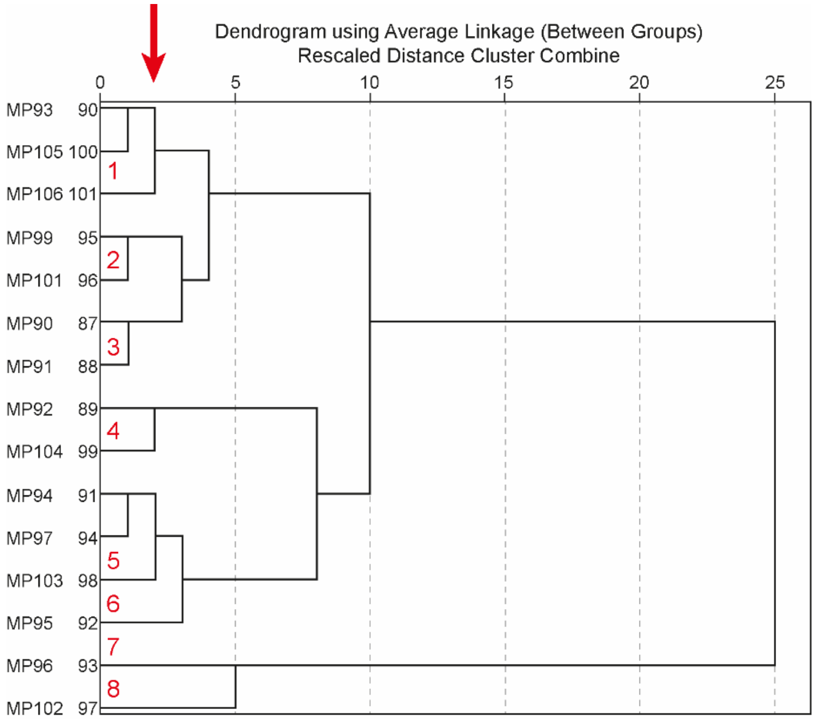

Fig. S1. Example dendrogram output from the hierarchical cluster analysis in SPSS with annotated cluster numbers.

Table S4. Cluster membership based on a rescaled distance of two on the example dendrogram in fig. S1.

**Cluster**	**Members**
1	MP93, MP105, MP106
2	MP99, MP101
3	MP90, MP91
4	MP92, MP104
5	MP94, MP97, MP103
6	MP95
7	MP96
8	MP102

Exploratory hierarchical cluster analysis showed that a rescaled distance of two on the dendrogram provided 21 clusters and therefore K-means analysis was run using this value. Intra-cluster isotope ranges in this model were in some instances far too heterogeneous to map onto single biosphere zones and some cases were very distant from cluster centres (maximum distance of 1.329 from centroid). Using a rescaled distance of one on the dendrogram provided 38 clusters, many of which were too similar and could therefore represent the same region. This model therefore comprised too many clusters. The model was reiterated and a K value of 24 fitted the dataset best. This had a maximum case distance from the cluster centre of 0.847 and almost all clusters were both sufficiently distinct and homogenous to represent single biosphere regions based on current mapping data. In some instances, $\delta^{34}S$ values were too wide to be likely to derive from a single region and the model therefore demonstrates a minimum of 24 distinct zones of provenance based on the three isotope proxies. The K value of 24 was deemed optimal, as increasing K yielded multiple clusters that could map on to the same biosphere region. The 24 clusters are summarised in terms of their membership, maximum distance from the cluster centre and the isotope value ranges in Table S5. Each sample's cluster membership is noted in Table S6 along with its distance from the cluster centre. The members in each cluster are listed in Table S7. In some instances the range of $\delta^{34}S$ values are broader than might be expected from a single location.

Current methods do not have the capability to demonstrate the number of regions represented in the dataset, and in any case, how one defines a region is open to question. It is highly likely that substantially more than 24 geographical regions are represented, but that some cannot be distinguished using the three isotopic indices, in spite of being in different parts of Britain. Similarly, in some instances different clusters may be distinct in terms of the three isotope proxies, but could represent adjacent regions, perhaps even comprising parts of the same community that lived, for example, on two sides of a geological boundary.

Table S5. Characterization of the clusters in the *K* = 24 model, showing membership (*n*) at each site, isotope value ranges, and the maximum distance from the cluster center among members.

Cluster	Durrington Walls (n)	Mount Pleasant (n)	WKPE (n)	Marden (n)	$^{87}Sr/^{86}Sr$	$\delta^{34}S$	$\delta^{18}O$	Max distance from centroid
1	3	0	1	1	0.7082-0.7088	13.0-15.4	27.0-27.6	0.440
2	2	0	2	0	0.7117-0.7127	15.6-16.4	24.4-25.4	0.551
3	1	0	0	0	0.7149	12.8	25.2	0.000
4	1	1	0	1	0.7094-0.7104	2.8-7.0	23.2-23.7	0.640
5	3	0	0	1	0.7106-0.7119	7.4-10.9	23.6-24.3	0.738
6	4	0	0	0	0.7092-0.7097	6.6-10.8	24.7-25.4	0.630
7	4	0	1	0	0.7087-0.7099	10.0-12.7	27.2-27.7	0.490
8	0	2	0	0	0.7084-0.7088	11.8-14.1	23.6-24.1	0.401
9	1	0	0	0	0.7148	14.2	26.5	0.000
10	6	0	0	0	0.7092-0.7107	7.1-9.2	26.2-26.7	0.751
11	3	0	0	1	0.7115-0.7135	10.7-14.2	25.3-25.9	0.633
12	3	0	0	0	0.7111-0.7116	11.7-12.8	26.3-27.2	0.535
13	18	2	2	0	0.7090-0.7103	11.7-16.2	25.6-26.9	0.689
14	1	0	0	0	0.7080	19.6	27.2	0.000
15	8	0	0	0	0.7085-0.7094	2.9-5.5	24.6-26.1	0.768
16	4	0	0	0	0.7080-0.7088	5.8-8.7	26.5-27.0	0.453
17	6	3	1	0	0.7098-0.7113	11.9-16.2	24.7-25.6	0.763
18	8	3	3	0	0.7080-0.7094	13.8-18.8	25.0-26.4	0.847
19	1	0	0	1	0.7166-0.7172	11.5-11.6	25.2-25.9	0.316
20	2	1	1	0	0.7097-0.7109	5.7-7.0	24.4-25.6	0.669
21	0	0	1	1	0.7131-0.7141	6.5-8.4	23.9-24.4	0.445
22	1	0	0	0	0.7097	-1.6	25.3	0.000
23	6	0	2	0	0.7083-0.7091	9.1-12.3	25.5-26.9	0.790
24	0	3	1	0	0.7104-0.7114	15.9-18.2	23.9-24.8	0.463

Table S6. Results of *K* = 24 cluster analysis ordered by sample number, showing cluster membership for each sample and its distance from the cluster centre.

Sample no.	Cluster	Distance	Sample no.	Cluster	Distance	Sample no.	Cluster	Distance
DW01	18	0.644	DW42	7	0.219	DW86	13	0.386
DW02	13	0.600	DW43	15	0.719	DW87	13	0.706
DW03	23	0.730	DW44	13	0.665	DW88	13	0.689
DW04	13	0.431	DW45	12	0.535	DW89	20	0.554
DW05	5	0.448	DW46	10	0.152	MP90	13	0.368
DW06	6	0.378	DW47	15	0.605	MP91	13	0.521
DW07	7	0.417	DW48	16	0.288	MP92	8	0.401
DW08	18	0.796	DW49	17	0.719	MP93	18	0.149
DW09	18	0.305	DW50	23	0.790	MP94	24	0.461
DW10	10	0.751	DW51	16	0.453	MP95	17	0.763
DW11	11	0.311	DW52	13	0.540	MP96	4	0.640
DW12	17	0.188	DW53	23	0.093	MP97	24	0.463
DW13	13	0.456	DW54	13	0.632	MP99	17	0.613
DW14	2	0.450	DW56	12	0.330	MP101	17	0.460
DW15	15	0.717	DW57	18	0.180	MP102	20	0.553
DW16	16	0.381	DW58	11	0.552	MP103	24	0.426
DW17	17	0.370	DW59	7	0.490	MP104	8	0.401
DW18	15	0.309	DW60	3	0.000	MP105	18	0.170
DW19	1	0.440	DW61	18	0.397	MP106	18	0.847
DW20	20	0.462	DW62	6	0.630	WK108	1	0.290
DW21	15	0.339	DW63	7	0.334	WK110	13	0.461
DW22	10	0.277	DW64	23	0.637	WK111	13	0.337
DW23	23	0.539	DW65	10	0.291	WK112	23	0.482
DW24	5	0.738	DW67	17	0.318	WK113	2	0.303
DW25	1	0.137	DW68	6	0.246	WK114	20	0.669
DW26	10	0.325	DW69	13	0.529	WK115	18	0.369
DW27	17	0.536	DW70	4	0.473	WK116	7	0.373
DW28	12	0.497	DW71	6	0.587	WK117	23	0.337
DW29	1	0.230	DW72	15	0.379	WK118	21	0.455
DW30	18	0.468	DW73	22	0.000	WK119	24	0.452
DW31	19	0.316	DW74	15	0.768	WK120	18	0.757
DW32	13	0.584	DW75	18	0.557	WK121	2	0.318
DW33	13	0.542	DW77	5	0.405	WK122	17	0.446
DW34	13	0.356	DW78	13	0.510	WK123	18	0.291
DW35	13	0.674	DW79	15	0.589	MD124	4	0.537
DW36	13	0.081	DW80	11	0.574	MD126	19	0.316
DW37	10	0.135	DW81	13	0.684	MD128	11	0.633
DW38	2	0.551	DW82	23	0.082	MD129	21	0.455
DW39	17	0.292	DW83	16	0.317	MD130	5	0.647
DW40	9	0.000	DW84	18	0.400	MD131	1	0.384
DW41	13	0.539	DW85	14	0.000			

Table S7. Results of the *K* = 24 cluster model, showing sample membership for each cluster.

Cluster	Members									
**1**	DW19	DW25	DW29	WK118						
**2**	DW14	DW38	WK113	WK121						
**3**	DW60									
**4**	DW70	MP96	MD124							
**5**	DW05	DW24	DW77	MD130						
**6**	DW06	DW62	DW68	DW71						
**7**	DW07	DW42	DW59	DW63	WK116					
**8**	MP92	MP104								
**9**	DW40									
**10**	DW10	DW22	DW26	DW37	DW46	DW65				
**11**	DW11	DW58	DW80	MD128						
**12**	DW28	DW45	DW56							
**13**	DW02	DW04	DW13	DW32	DW33	DW34	DW35	DW36	DW41	DW44	DW52
**13 (cont)**	DW54	DW69	DW78	DW81	DW86	DW87	DW88	MP90	MP91	WK110	WK111
**14**	DW85									
**15**	DW15	DW18	DW21	DW43	DW47	DW72	DW74	DW79		
**16**	DW16	DW48	DW51	DW83						
**17**	DW12	DW17	DW27	DW39	DW49	DW67	MP95	MP99	MP101	WK122	
**18**	DW01	DW08	DW09	DW30	DW57	DW61	DW75	DW84	MP93	MP105	MP106
**18 (cont)**	WK115	WK120	WK123							
**19**	DW31	MD126								
**20**	DW20	DW89	MP102	WK114						
**21**	WK118	MD129								
**22**	DW73									
**23**	DW03	DW23	DW50	DW53	DW64	DW82	WK112	WK117		
**24**	MP94	MP97	MP103	WK119						

Radiogenic values

Table S8 presents multi-isotope data for individuals with highly radiogenic (>0.7131) $^{87}Sr/^{86}Sr$ values.

Table S8. Samples with highly radiogenic (>0.7131) $^{87}Sr/^{86}Sr$ values.

Sample	$^{87}Sr/^{86}Sr$	$\delta^{34}S$ (‰)	$\delta^{18}O$ (‰)
DW31	0.7172	11.6	25.19
MD126	0.7166	11.5	25.85
DW60	0.7149	12.8	25.20
DW40	0.7148	14.2	26.46
MD129	0.7141	6.5	23.88
MD128	0.7135	10.7	25.76
WK121	0.7133	15.6	25.06
WK118	0.7131	8.4	24.41
